# Evolved *Bmp6* enhancer alleles drive spatial shifts in gene expression during tooth development in sticklebacks

**DOI:** 10.1093/genetics/iyab151

**Published:** 2021-09-23

**Authors:** Mark D. Stepaniak, Tyler A. Square, Craig T. Miller

**Affiliations:** Department of Molecular and Cell Biology, University of California, Berkeley, Berkeley, CA 94720, USA

**Keywords:** enhancer, *cis*-regulation, evolution, transgene, transgenesis, insulator, fish, stickleback, development, tooth

## Abstract

Mutations in enhancers have been shown to often underlie natural variation but the evolved differences in enhancer activity can be difficult to identify *in vivo*. Threespine sticklebacks (*Gasterosteus aculeatus*) are a robust system for studying enhancer evolution due to abundant natural genetic variation, a diversity of evolved phenotypes between ancestral marine and derived freshwater forms, and the tractability of transgenic techniques. Previous work identified a series of polymorphisms within an intronic enhancer of the *Bone morphogenetic protein 6* (*Bmp6*) gene that are associated with evolved tooth gain, a derived increase in freshwater tooth number that arises late in development. Here, we use a bicistronic reporter construct containing a genetic insulator and a pair of reciprocal two-color transgenic reporter lines to compare enhancer activity of marine and freshwater alleles of this enhancer. In older fish, the two alleles drive partially overlapping expression in both mesenchyme and epithelium of developing teeth, but the freshwater enhancer drives a reduced mesenchymal domain and a larger epithelial domain relative to the marine enhancer. In younger fish, these spatial shifts in enhancer activity are less pronounced. Comparing *Bmp6* expression by *in situ* hybridization in developing teeth of marine and freshwater fish reveals similar evolved spatial shifts in gene expression. Together, these data support a model in which the polymorphisms within this enhancer underlie evolved tooth gain by shifting the spatial expression of *Bmp6* during tooth development, and provide a general strategy to identify spatial differences in enhancer activity *in vivo*.

## Introduction

The process of development is largely orchestrated by developmental regulatory genes whose spatial and temporal patterns of transcription are controlled by enhancers, *cis*-regulatory elements that bind transcription factors and promote transcription of target genes (Furlong and Levine 2018; Gasperini *et al.* 2020). Most developmental regulatory genes are pleiotropic, and function repeatedly at different times and in different tissues during development (Sabarís *et al.* 2019). Thus, mutations in enhancers of developmental regulatory genes are often more tolerated than coding sequence mutations due to having fewer pleiotropic effects, as the impacts of enhancer mutations are more likely to be restricted in time and/or space, compared to the anatomically more widespread impacts of coding mutations (Carroll 2008). The importance of enhancers in regulating morphological evolution, natural variation, and disease phenotypes in humans is well established (Rebeiz and Tsiantis 2017; Rickels and Shilatifard 2018). However, a growing need has emerged for methods and approaches to compare the activity of molecularly divergent enhancer alleles.

*Cis*-regulatory changes have been shown to underlie the evolution of multiple morphological traits in threespine stickleback fish (*Gasterosteus aculeatus*). Threespine sticklebacks live in both marine and freshwater environments in the Northern Hemisphere, repeatedly forming populations in rivers, streams, ponds, and lakes from ancestral marine populations (Bell and Foster 1994; McKinnon and Rundle 2002). Following a freshwater colonization event, a suite of traits has been observed to typically evolve such as reduction in armor (Bell and Foster 1994; Cresko *et al.* 2004; Colosimo *et al.* 2005) and changes in body shape (Walker 1997; Walker and Bell 2000; Albert *et al.* 2008; Reid and Peichel 2010). Other traits that typically evolve major differences are those associated with feeding morphology, likely an adaptation to different diets of larger prey in freshwater environments relative to marine ancestral environments (Hagen 1967; Gross and Anderson 1984; Lavin and McPhail 1986; Schluter and McPhail 1992; Bell and Foster 1994). High-resolution genetic mapping studies have implicated *cis*-regulatory changes as underlying several phenotypes that have evolved in freshwater, including the reduction of armor plates (Colosimo *et al.* 2005; O’Brown *et al.* 2015; Indjeian *et al.* 2016; Archambeault *et al.* 2020), pelvic spines (Chan *et al.* 2010), and pigmentation (Miller *et al.* 2007), and increases in branchial bone length (Erickson *et al.* 2018), and pharyngeal tooth number (Cleves *et al.* 2014, 2018).

Tooth development is orchestrated by reciprocal signaling between dental epithelium and dental mesenchyme (Balic and Thesleff 2015). Tooth competence initially resides in the dental epithelium and is subsequently transferred to dental mesenchyme (Lumsden 1988). These two tissues coordinate tooth morphogenesis, with inner dental epithelial cells generating ameloblasts that secrete enamel and/or enameloid that covers the outside of the tooth, and mesenchymal cells generating odontoblasts that secrete the dentine that comprises the inner mineralized part of a tooth. Within the inner dental epithelium, future tooth cusp regions locally express different patterns of growth factors and are called enamel knots (Thesleff *et al.* 2001). Overall, the roles of dental epithelia and mesenchyme during tooth development are poorly understood. For example, in some experiments, dental mesenchyme positively regulates tooth number (Cai *et al.* 2007) while in other experiments dental mesenchyme negatively regulates tooth number (Munne *et al.* 2009).

Most work on tooth development has been done in monophyodont rodents that do not replace teeth. In polyphyodont vertebrates such as fish, teeth are regenerated throughout adult life. Most fish have two sets of jaws, both of which constantly regenerate teeth: an oral jaw in their first or mandibular segment and pharyngeal jaws usually in the seventh pharyngeal segment (Fraser *et al.* 2009; Ellis *et al.* 2016). Oral and pharyngeal teeth develop and replace similarly, and in sticklebacks, are morphologically indistinguishable and display similar patterns of gene expression (Ellis *et al.* 2016). Little is known about the molecular genetic circuitry regulating tooth replacement, but genetic analysis in sticklebacks has provided a powerful system to address this question.

Increases in pharyngeal tooth number have evolved independently in multiple freshwater stickleback populations (Ellis *et al.* 2015). Comparing lab-reared marine fish and freshwater fish from the benthic (bottom-dwelling) population of Paxton Lake revealed that a divergence in tooth number occurs late in development (around ∼20 mm total length, when fish are juveniles and about half of their adult length). This difference in tooth number continues to increase and becomes more significantly different at adult stages (Cleves *et al.* 2014). Quantitative trait loci (QTL) mapping identified a large effect QTL that underlies this evolved tooth gain. An F_2_ cross between a low-toothed Japanese marine fish and a high-toothed benthic Paxton Lake freshwater fish identified a QTL peak on chromosome 21 that explained approximately 30% of the variance in tooth number within the cross (Miller *et al.* 2014). The peak contained the candidate gene *Bone morphogenetic protein 6* (*Bmp6*) which is dynamically expressed in developing teeth. *In situ* hybridization revealed *Bmp6* expression early in inner dental epithelium, as well as in underlying dental mesenchyme, followed by a decrease in expression in the epithelium before the tooth finally erupts into a functional tooth (Cleves *et al.* 2014; Ellis *et al.* 2016). Allele-specific expression (ASE) experiments identified *cis-*regulatory changes in *Bmp6*. In tooth tissue from F_1_ hybrids of high-toothed Paxton benthic fish and low-toothed marine fish, a 1.4-fold decrease in *Bmp6* expression from the high-tooth freshwater Paxton benthic allele compared to the marine allele was reported (Cleves *et al.* 2014). Work in mice and fish has demonstrated an essential role for BMPs in developing teeth (Vainio *et al.* 1993; Bei *et al.* 2000; Wang *et al.* 2012; Jia *et al.* 2013; Cleves *et al.* 2018), suggesting a possible causative role of *Bmp6* in evolved tooth gain.

Further refinement of the QTL interval identified a haplotype containing 10 single nucleotide polymorphisms (SNPs) within intron 4 of *Bmp6* that vary concordantly with the presence or absence of the tooth QTL (Cleves *et al.* 2018). These variable positions define a high-tooth associated haplotype and low-tooth associated haplotype from the Paxton benthic freshwater and marine alleles, respectively. Six core SNPs lie within 468 bases upstream of the previously described minimally sufficient *Bmp6* intron 4 tooth enhancer (Supplementary Figure S1) (Cleves *et al.* 2018). We hypothesized that these core QTL-associated SNPs are modifying the spatial and/or temporal activity of the adjacent tooth enhancer.

Comparing expression patterns of two different alleles of an enhancer through reporter constructs in an organismal context presents three major problems: (1) comparisons of enhancer variants integrated in two different organisms are difficult to fully control for developmental time and genetic background differences, (2) aspects of reporter expression may in part reflect genomic integration site rather than actual enhancer activity, and (3) different fluorophores are known to have different physical properties (Cranfill *et al.* 2016) and thus reporter gene differences with different fluorophores might reflect differences in fluorophore brightness, stability, *etc*. instead of differences in enhancer activity. A single bicistronic transgenic construct that contains both enhancer/reporter pairings could address the first problem by providing a comparison within the same animal (and thus both enhancers being compared are at the same stage and in the same genotype). Furthermore, a single bicistronic construct simultaneously reduces the number of genomic integration sites to one and thus reduces position effects, partially addressing the second problem. The placement of a genetic insulator between the enhancer-reporter pairings can reduce cross talk of an enhancer with the opposite paired reporter, creating a more accurate expression profile. Genetic insulators have been shown to be effective in zebrafish (Bessa *et al.* 2009; Shimizu and Shimizu 2013). A second alternative approach to a single bicistronic transgene is the use of doubly transgenic two-color lines that include both marine and freshwater enhancers paired with different reporters as parts of separate transgenes. This approach addresses the first problem by having both enhancers in the same animal. With this doubly transgenic two-color line approach, enhancers can be tested with reciprocal pairings (*i.e.*, multiple transgenic reporter lines with different enhancers driving different fluorophores), to control for possible position effects and possible fluorophore differences. Here we use transgenic reporter assay experiments to test the hypothesis that the marine and freshwater *Bmp6* intron 4 enhancers have different spatial and/or temporal activity in developing fish embryos, larvae, and adults. We tested this hypothesis in two ways: first, by using a bicistronic enhancer transgene to compare activities of two enhancers in the same fish, and second, by comparing doubly transgenic two-color fish in which the marine and freshwater enhancers drive different fluorophores from different genomic integrations. Lastly, we tested whether the spatial shifts in enhancer activity between marine and freshwater enhancers are also observed for endogenous patterns of *Bmp6* expression during tooth development in marine and freshwater fish.

## Materials and methods

### Animal statement

All animal work was approved by UCB animal protocol #AUP-2015-01-7117-2. Fish were reared as previously described (Erickson *et al.* 2014).

### Insulator containing bicistronic construct

Gibson assembly was used to create bicistronic constructs to determine insulator efficiency in sticklebacks. Two enhancers with distinct expression domains were used: a 1.3-kb fragment from intron 4 of *Bmp6* (Cleves *et al.* 2018) and the stickleback ortholog of the R2 enhancer for *Col2a1a*, first identified in zebrafish and previously shown to drive similar embryonic expression in sticklebacks (Dale and Topczewski 2011; Erickson *et al.* 2016). These two enhancers were placed on opposite sides of a genetic insulator, each with a different reporter gene, either mCherry (mCh) or enhanced GFP (eGFP). The mouse tyrosinase GAB (Guanine-rich sequence with A and B boxes) insulator was PCR amplified from the 2pC_GS plasmid (Bessa *et al.* 2009), while the R2 *Col2a1a* enhancer was PCR amplified from a previously used reporter plasmid (Erickson *et al.* 2016). The intron 4 enhancer of *Bmp6* was PCR amplified from a reporter plasmid containing either the freshwater allele from the benthic Paxton Lake population or the allele from the Little Campbell marine population (Cleves *et al.* 2018). All enhancers were PCR amplified simultaneously with the *Hsp70l* promoter as a single amplicon. eGFP and mCh were amplified from previously used reporter plasmids (O’Brown *et al.* 2015). Primers used and assembly steps are listed in the Supplemental Methods. All components were combined using a Gibson assembly reaction (New England Biolabs ref # E2611L) following the manufacturer’s protocol and transformed into XL1 blue competent cells (Agilent). Transformed cells were grown on ampicillin-containing LB plates and colony inserts were sequence verified by colony PCR. Positive colonies were used to start 50 ml cultures, which were grown overnight. Plasmids were then isolated by Qiagen midi-prep (#12145) and inserts fully verified by Sanger sequencing.

Tol2 transposase mRNA was transcribed using the plasmid pCS2-TP (Kawakami 2004) that had been linearized with *Not*I. The linear plasmid was used as template for *in vitro* transcription using the mMessage SP6 kit (#AM1340). The resulting mRNA was purified using Qiagen RNeasy columns (#74104). Transgene plasmids were co-injected with Tol2 mRNA into newly *in vitro* fertilized one-cell embryos as described (Erickson *et al.* 2016). Approximately 200 ng of plasmid in 1 µl was combined with 1 µl of 2M KCl, 0.5 µl of 0.5% phenol red, and approximately 1 µl of 350 ng/µl of Tol2 transposase mRNA, with water added to a final volume of 5 µl, yielding a total concentration of ∼40 ng/µl of plasmid and 70 ng/µl of mRNA. Embryos were generated from Rabbit Slough (RABS; Alaska) marine fish, and lines established and maintained by crossing to lab-reared fish from this same population.

### Generation of single color and doubly transgenic two-color reporter lines

The previously described ∼1.3 kb *Bmp6* intron 4 tooth enhancer (Cleves *et al.* 2018) was amplified from a Paxton Lake benthic fish and Little Campbell marine fish (Supplementary Figure S1) using the primer pairs MDS35/36 (GCCGGCTAGCGAGAGCATCCGTCTTGTGGG/GCCGGGATCCAGAGTCCTGATGGCCTCTCC) to create reporter plasmids containing the positive orientation (*i.e.*, same 5′ to 3′ orientation as in endogenous locus) of the enhancer relative to the reporter gene or MDS27/28 (GCCGGCTAGCAGAGTCCTGATGGCCTCTCC/GCCGGGATCCGAGAGCATCCGTCTTGTGGG) to create reporter plasmids containing the negative orientation [*i.e.*, the opposite 5′ to 3′ orientation as in the endogenous locus, and possibly more similar to the orientation that an enhancer 3′ to the promoter (*e.g.*, an enhancer in intron 4) would be after looping to contact the promoter] of the enhancer. The fragments were then cloned in both possible 5′ to 3′ orientations into a Tol2 reporter construct upstream of the zebrafish *Hsp70l* promoter and either eGFP or mCh using *BamH*I and *Nhe*I in the previously generated reporter constructs. Fish that were transgenic for both the marine and the freshwater reporter alleles were generated in one of two ways: (1) crossing of stable lines each containing a single transgene and (2) injection of one reporter construct into a stable transgenic line of the opposite (*i.e.*, different population and fluorophore) allele.

### Detecting enhancer activity by fluorescence microscopy

Enhancer activity of the transgenic constructs was imaged by fluorescence microscopy. Previous work demonstrated a *cis*-regulatory difference in *Bmp6* expression between marine and freshwater alleles, with the difference arising late in development (Cleves *et al.* 2014). As both a divergence in tooth number attributed to the QTL and allele ASE differences arise late in development, post-20 mm total length (Cleves *et al.* 2014; 2018), reporter positive fish were dissected at total lengths pre- and post-tooth number divergence (20 mm total length) as previously described (Ellis and Miller 2016). Tooth plates were then fixed in 4% PFA (paraformaldehyde) in 1× phosphate-buffered saline (PBS) for 60 min, washed through a graded series of 3:1, 1:1, 1:3 PBS and glycerol solutions into 100% glycerol, flat-mounted, and imaged. Comparisons were made across the different alleles and orientations on a Leica M165FC dissecting microscope with filters GFP1 (#10447447) and RhodB (#10447360), and a Leica DM2500 compound microscope with filters GFP (#11532366) and TX2 (#11513885). To compare enhancer activity in fish before and after tooth divergence, ventral tooth plates and dorsal tooth plates were imaged and enhancer activity was assessed in the dental epithelium and mesenchyme of each tooth, in each of three pre-divergence sized fish (between 16 and 18.5 mm total length) and three post-divergence sized fish (between 30 and 48 mm total length) in two different sets of integrations and enhancer/reporter pairings. If the QTL-associated SNPs are responsible for the QTL peak and therefore tooth number differences observed late in development, as well as the ASE differences, we would expect the enhancers to have different activity in >20 mm fish compared to <20 mm fish. We would also expect the enhancers to have similar activity earlier in development, when ASE was not significantly different between the freshwater and marine alleles (Cleves *et al.* 2014).

### Quantification of enhancer activity differences across tooth development

As we hypothesized that the QTL-associated intronic polymorphisms result in differential enhancer activity in the dental mesenchyme and/or epithelium, we characterized enhancer activity in both tissues across multiple tooth plates. The stage of each tooth was scored as either early (late cap to early bell stages in which mesenchyme has condensed under the epithelium but no mineralization has occurred), middle (mineralization of the forming tooth has started to occur, also called late bell stage), or late [a fully formed tooth has erupted, also called functional stage (Ellis *et al.* 2015)]. The activity for each enhancer allele was recorded as either present or absent in the epithelium (early and middle stages) and mesenchyme (all three stages). Additionally, we also recorded if either allele (marine or freshwater) drove more robust or extensive expression in each domain, indicating an allelic bias.

To quantify the expression domain sizes of marine and freshwater tooth enhancer alleles, we used the “measure” function in ImageJ (Schneider *et al.* 2012) on scaled fluorescence images of developing tooth germs. We measured the 2D (X/Y) mesenchymal domain areas for both the freshwater and marine alleles in three tooth germs each from 12 fish: one tooth germ for each of the three tooth stages (“early,” “middle,” and “late”), analyzed for both reporter construct orientations/fluorophore combinations, and assayed on fish of both <20 and >20 mm total length (for a total of 36 tooth germs, from 12 different fish). The epithelial measurements were taken using the same set of tooth germ images but excluding the “late” stage of tooth germ development because the epithelium becomes ruptured and degrades at this stage (for a total of 24 tooth germs, from 12 different fish).

To test if transgene construct orientation and fluorophore combination significantly affected expression domain size, Wilcoxon rank-sum two-tailed tests were used in R (R Core Team 2020) on mesenchymal and epithelial expression domain areas for marine and freshwater enhancer alleles at each tooth stage (*n* = 6 *vs* 6 teeth in all tests, combining fish from early and late fish stages, with each tooth from a different fish). To test if the freshwater allele drove a reduced mesenchymal area and an expanded epithelial domain relative to the marine allele, Wilcoxon signed-rank one-tailed tests were used in R, paired for each tooth germ assessed (*n* = 12 for each tooth germ stage, with each tooth from a different fish). To test if reduction in the mesenchymal area or expansion in epithelial area for the freshwater allele relative to the marine allele were more significant at >20 mm fish stages than <20 mm fish stages, we used Wilcoxon rank-sum one-tailed tests in R (*n* = 6 teeth for each tooth germ stage at each fish stage, with each tooth from a different fish).

### *In situ* hybridization on sections

Stickleback adult (∼40 mm standard length) pharyngeal tissues were prepared, sectioned, and assayed by *in situ* hybridization (ISH) in parallel to compare the spatial distribution of *Bmp6* mRNA. Adults derived from marine (RABS) and freshwater [Paxton Benthic (PAXB)] populations were euthanized, and their pharyngeal tissues were fixed overnight in 4% formaldehyde (Sigma P6148) in 1× PBS at 4°C with heavy agitation, washed 3× for 20 min with PBST (1x PBS, 0.1% Tween) on a nutator, then decalcified for 5 days in 20% ethylenediaminetetraacetic acid (EDTA, pH 8.0) at room temperature on a nutator. Marine and freshwater fish were always collected and prepared in parallel such that all storage and preparation intervals were equivalent. The ISH for *Bmp6* was carried out as described previously (Square *et al.* 2021), with some modifications to ensure maximally comparable assays were performed on marine and freshwater samples in parallel. A previously published *Bmp6* riboprobe was used in this study (Cleves *et al.* 2014; Square *et al.* 2021). The *Bmp6* riboprobe was synthesized with digoxygenin-labeled UTP and added at a concentration of ∼300 ng/ml in 20 ml of hybridization buffer, split between two different LockMailer slide containers (Sigma-Aldrich), and agitated overnight in a rotating hybridization oven at 67°C. Slides from marine and freshwater fish were cohoused in the hybridization buffers to ensure equal exposure to the riboprobe between marine and freshwater samples. Hybridization buffer washes, blocking, and antibody incubation steps were as previously described (Square *et al.* 2021). Signal development was carried out for 2, 3, or 7 days to visualize mRNA localization. Marine and freshwater slides were developed in parallel (in the same solutions, in the same LockMailer containers), and only those sections that experienced the same coloration reaction were compared (*i.e.*, we only directly compared sections that were prepared in parallel). To prepare slides for imaging, they were counterstained with DAPI, rinsed then washed 3× for 5+ min with deionized H_2_O, coverslipped with deionized H_2_O, and imaged on a Leica DM2500 microscope. The procedure outlined in this section was replicated three times, each replication used two marine and two freshwater adults, for a total of *n* = 6 fish from each background.

## Results

### Two ways to compare enhancers in transgenic fish

We used two strategies to compare enhancer alleles in the same transgenic fish. First, we used a single bicistronic construct with a genetic insulator separating two enhancer/reporter pairs. Second, we used two separate transgenic constructs, independently integrated in the same fish line and each containing a single enhancer allele (marine or freshwater, Supplementary Figure S1) with a distinct fluorescent reporter (eGFP or mCh), to generate doubly transgenic two-color fish.

### Insulator efficiency in F_0_ fish

To test the first strategy of a bicistronic construct separated by an insulator, a bicistronic construct was generated using two enhancers that drive expression in non-overlapping domains. In sticklebacks, the *Col2a1a R2* enhancer drives expression in the developing notochord with expression seen by the third day post fertilization (dpf) (Erickson *et al.* 2016). By 8 dpf, we observed *R2* reporter expression in the developing craniofacial skeleton, including Meckel’s cartilage, the hyosympletic, and the ceratohyal (Supplementary Figure S2), similar to the reported enhancer activity in zebrafish (Dale and Topczewski 2011). The *Bmp6* intron 4 tooth enhancer has not been reported to drive expression in the domains seen in the *R2 Col2a1a* enhancer. In addition, the previously described tooth and early fin domains (Cleves *et al.* 2018), as well as the presently described late fin domains, are not domains in which the *Col2a1a* enhancer has been observed to drive expression. Thus, to our knowledge, these two enhancers drive distinct and non-overlapping expression domains within these embryonic and larval tissues, providing multiple locations that can test for insulation within the construct.

**Figure 1:**
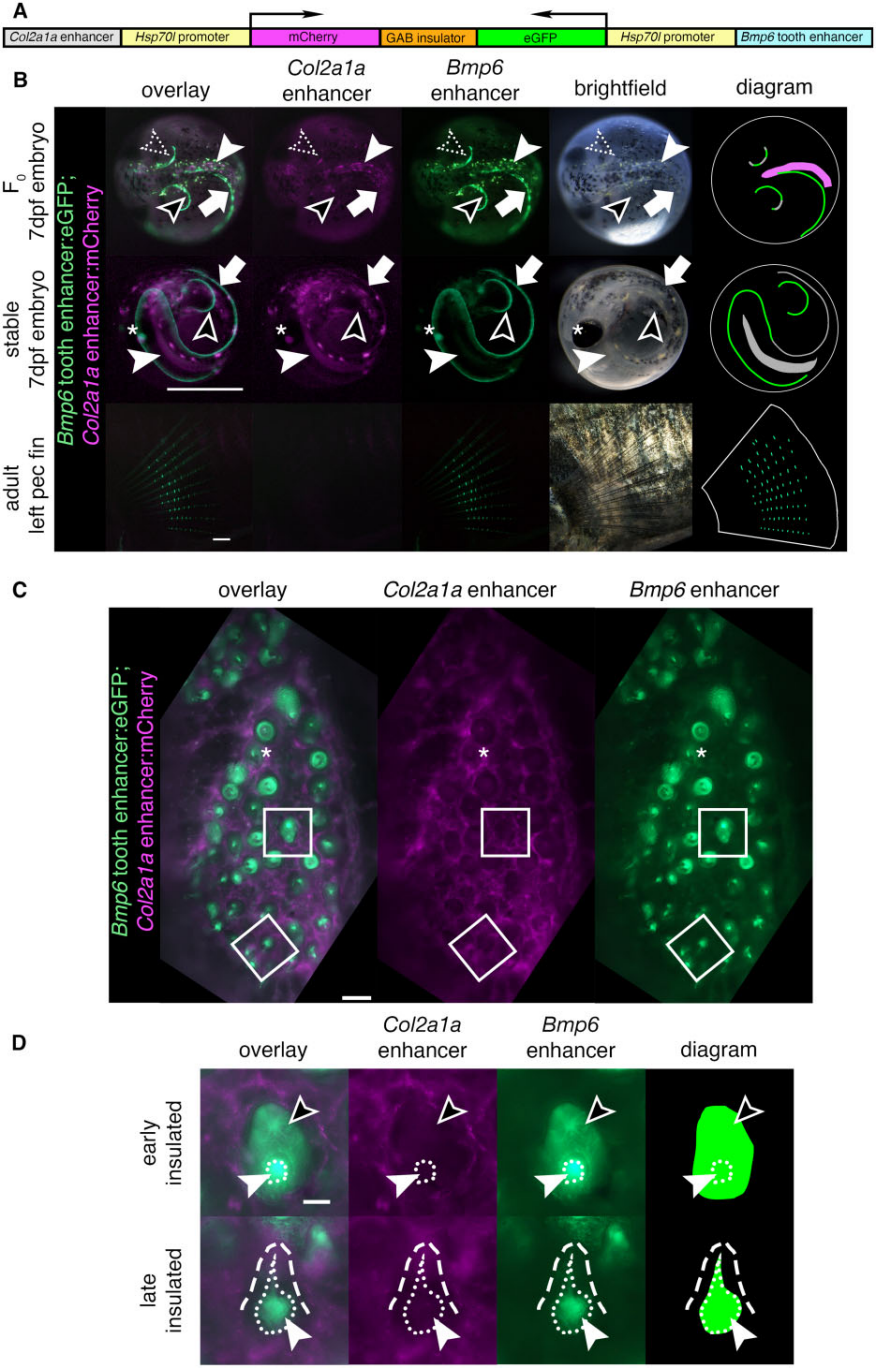
An insulated bicistronic construct reports separate expression patterns from two different enhancers. (A) Bicistronic construct with a *Col2a1a* enhancer and *Hsp70l* promoter driving mCh and the freshwater *Bmp6* intronic tooth enhancer and *Hsp70l* promoter driving eGFP, separated by the mouse tyrosinase insulator (GAB). (B) Transgenic fish show a separation of domains in red and green overlay, red channel only, green channel only, brightfield, and diagram (left to right). Top: In 7 days post fertilization (dpf) F_0_ embryos (dorsal view), insulation was observed in some but not all domains. Both mCh and eGFP were observed in the same area in the right pectoral fin (dotted arrowhead), indicating incomplete or failed separation of domains, while in other areas of the pectoral fin only eGFP was observed (black arrowhead). Within the notochord (solid white arrowhead), only mCh was observed, while in the median fin (white arrow) only eGFP was observed, indicating insulation in both domains. Middle: In 7 dpf stable F_1_ embryos (lateral view), only eGFP was observed in the pectoral fins (black arrowhead) indicating successful insulation in those domains, while both fluorophores were detected in the median fin (white arrow) and in the notochord (solid white arrowhead) indicating a lack of insulation. Both fluorophores were detected in the lens of the eye (asterisk), a domain driven by the *Hsp70l* promoter. Bottom: in adult pectoral fins (lateral view), eGFP but not mCh expression was detected. Diagram: schematic of eGFP and mCh expression in fins and notochord, with overlap shown in gray. Spheres trace the outline of the chorion (top, middle), and white lines trace the pectoral fin (bottom). (C and D) Dorsal pharyngeal tooth plate (C) and representative teeth of early and late stages (D) from adult stable transgenic fish. (C) Insulator effectiveness was observed with eGFP restricted to predicted tooth domains and mCh primarily present in the surrounding tissue. In some teeth, faint mCh appeared to be expressed in the dental mesenchyme (asterisk). (D) eGFP expression was detected in the dental mesenchyme (solid arrowhead and extent of mesenchyme as white dotted line) and dental epithelium (black arrowhead) of developing teeth, while mCh was expressed in the surrounding tissue (white dashed line outlines a mineralized tooth). Scale bars=1 mm (B), 100 µm (C), 25 µm (D). *n*=92 F_0_ embryos, >50 F_1_ embryos, >3 adult fish per time point and 3 teeth per fish for adult stage.

Three clutches were injected with a *Col2a1a* enhancer/*Bmp6* tooth enhancer bicistronic construct (Figure 1A) for a total of 228 injected embryos, of which 92 were scoreable at 7 dpf. Four domains (left and right pectoral fins, median fin fold, and notochord) were scored for insulation efficiency (0–2 for no to complete insulation, see Supplementary Figure S3 and Supplemental Methods). Across all domains, the average insulator score was 0.94 (Supplementary Table S1). Overall, the bicistronic construct using the mouse tyrosinase insulator element (GAB) moderately prevented reporter genes from being activated by nearby enhancers when placed between the elements. Within the same F_0_ fish, we observed both insulated and uninsulated domains, with insulation even varying within a domain (Figure 1B). For example, insulation was observed in the median fin and most of the left pectoral fin, but not within some regions of the right pectoral fin of a 7-dpf embryo in which both mCh and eGFP were observed. To control for enhancer/reporter pairing, the inverse construct was created, with the *Col2a1a* enhancer driving eGFP and the *Bmp6* tooth enhancer driving mCh. A total of 154 fish were injected across two clutches, with 30 surviving to 7 dpf that were scoreable, with an average score of 0.64 (Supplementary Table S2). Overall, both insulator constructs demonstrate the ability to drive some degree of separate expression domains of two enhancers concurrently, consistent with results reported in zebrafish that showed insulators can block enhancer-promoter crosstalk (Bessa *et al.* 2009).

### Insulator effectiveness in stable fish

Variation in insulator effectiveness across an individual F_0_ fish may be due to different genomic integrations of the bicistronic constructs. To determine the effectiveness of a single bicistronic transgene, F_0_ fish were outcrossed to create stable F_1_ individuals for the *Col2a1a R2*: mCh; *Bmp6* tooth enhancer: eGFP bicistronic construct. In 7 dpf F_1_ embryos, complete fin domains of the *Bmp6* enhancer were observed, with insulation apparent in some but not all domains (Figure 1B). In adults, *Bmp6* enhancer activity was observed in the intersegmental joints of fins (described below), however, no mCh was observed, suggesting effective insulation in that domain (Figure 1B). Insulator activity was also observed in pharyngeal teeth (Figure 1C). The *Bmp6* enhancer was observed to drive expression in the mesenchyme and inner dental epithelium of pharyngeal teeth (Figure 1D), consistent with previous reports. mCh was not observed in nearly all tooth domains, suggesting effective insulation in adult teeth. Thus, in stable transgenic adults, the insulator can separate the activity of the two enhancers, including within the dental epithelial and mesenchymal domains of the *Bmp6* enhancer.

### Bicistronic construct reveals spatial shifts in mesenchymal and epithelial activity of *Bmp6* enhancer alleles

**Figure 2:**
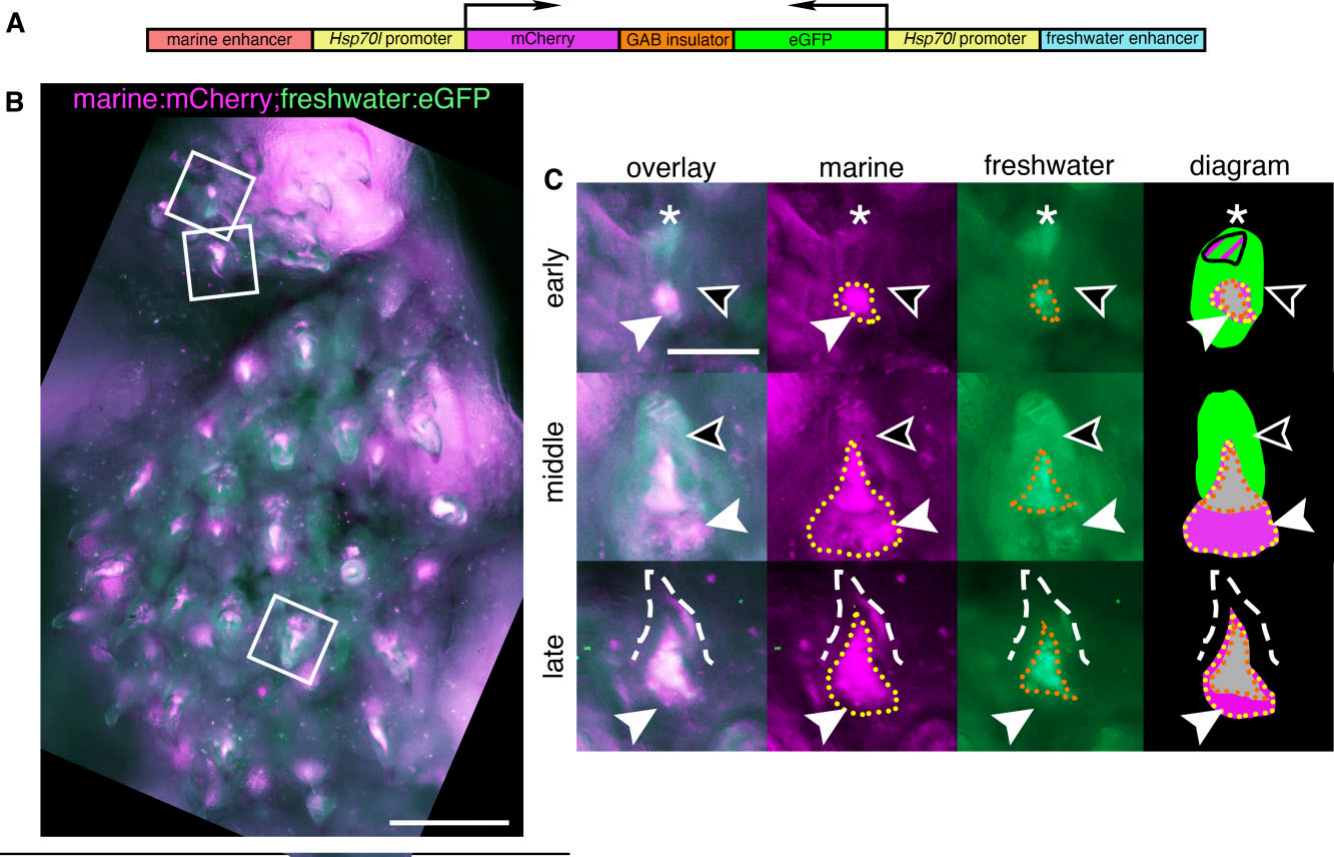
A bicistronic construct using a genetic insulator separates the expression domains of the marine and freshwater alleles of the *Bmp6* tooth enhancer. (A) Bicistronic construct with the marine allele of the intron 4 *Bmp6* enhancer/*Hsp70l* promoter driving mCh and the freshwater allele/*Hsp70l* promoter driving eGFP separated by the mouse tyrosinase GAB insulator. (B) Dorsal pharyngeal tooth plate from a fish transgenic with construct (A), and representative teeth (white boxes) from early, middle, and late stages (early bell, late bell, and functional, respectively) (C). Early: epithelium expressed eGFP throughout (black arrowhead) while a concentrated tip (asterisk) was observed to contain both marine and freshwater activity. In the mesenchyme (white arrowhead) the marine allele had a more robust and larger expression domain (yellow dotted line) compared to the freshwater allele (orange dotted line). Middle: epithelium had freshwater expression while the marine allele continued to drive more robust expression in the mesenchyme compared to the freshwater allele. Late: As in the other stages, the freshwater allele had a more restricted expression domain in mesenchyme of erupted mineralized teeth (dashed line). Diagram: summary of tooth epithelial and mesenchymal domains. Overlapping mesenchyme domain is gray, and expanded marine mesenchyme is marked with white arrowhead. Scale bars=200 µm (B), 50 µm (C). *n*=3 fish, 3 teeth per fish.

Since the GAB genetic insulator can block enhancer-promoter crosstalk in bicistronic constructs, a bicistronic construct with both the marine and freshwater alleles (Figure 2A) was used to create a stable line as a first test for enhancer activity differences. The marine allele, paired with mCh, appeared to drive a more robust mesenchymal domain compared to the freshwater allele (Figure 2, B and C). In contrast, within the inner dental epithelium, more GFP than mCh signal was detected, suggesting an expanded epithelial domain driven by the freshwater enhancer compared to the marine allele. Thus, in developing teeth from fish with this bicistronic transgene, the marine allele drove more expression in the mesenchyme while the freshwater allele drove more expression in the epithelium.

### Doubly transgenic fish confirm expanded freshwater epithelial *Bmp6* enhancer activity in post-divergence fish

**Figure 3:**
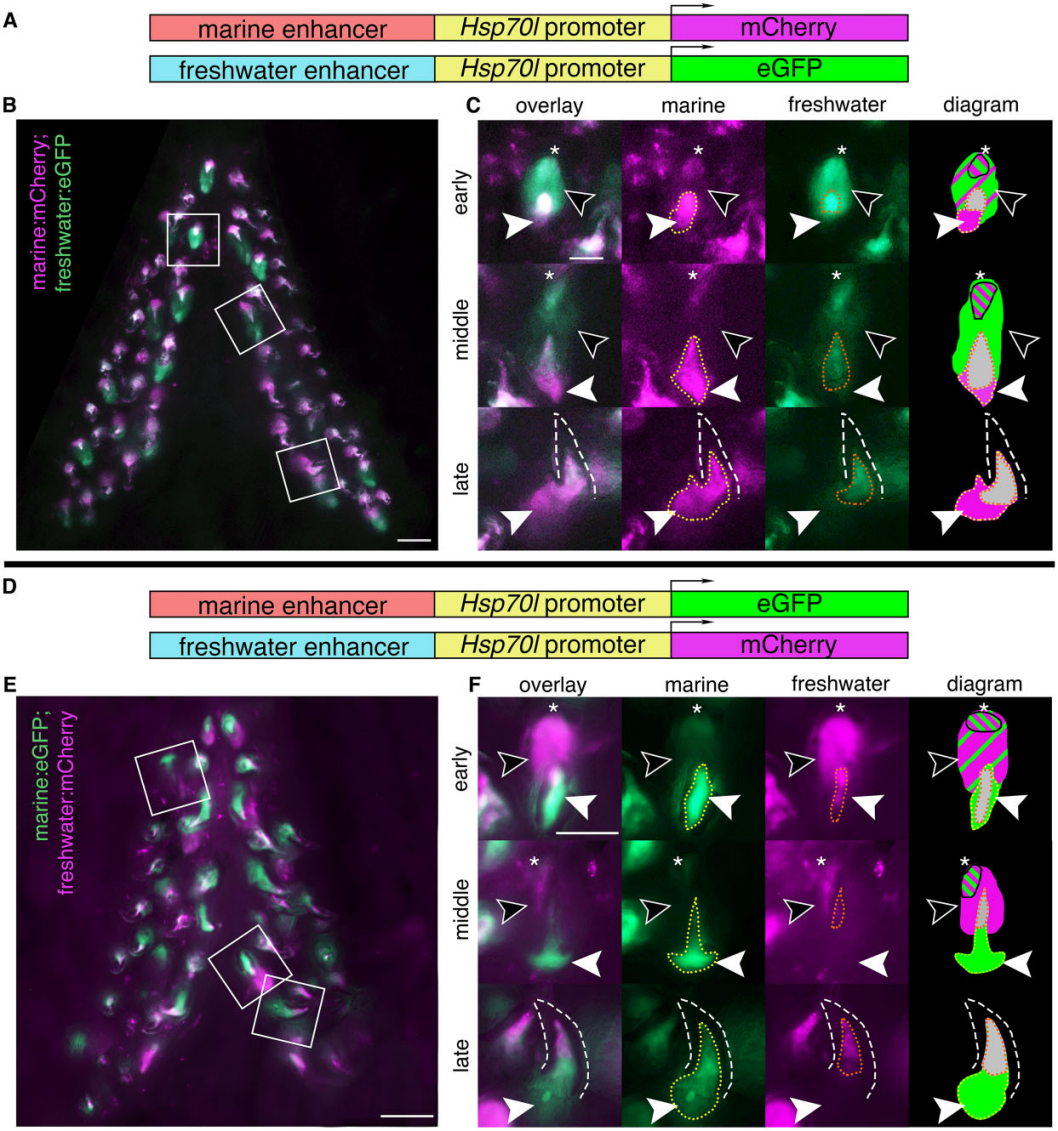
Reduced mesenchymal and expanded epithelial expression of freshwater enhancer relative to marine enhancer in developing ventral pharyngeal teeth. Ventral pharyngeal tooth plates from fish doubly transgenic for two alleles of the *Bmp6* intron 4 enhancer driving two different reporter genes (A, D): the marine enhancer driving mCh with the freshwater enhancer driving eGFP (B, C) and the marine enhancer driving eGFP with the freshwater enhancing driving mCh (E, F). Bilateral ventral pharyngeal tooth plates (B, E) are shown, next to representative teeth from three stages (C, F): early (early bell), middle (late bell), and late (functional) highlighted by white boxes in B, E. (C, F) Early: freshwater and marine enhancer drove expression in the epithelium (black arrowheads), with concentrated expression in the tip (asterisk), and more overall epithelial expression from the freshwater enhancer. Both enhancers also drove expression in the mesenchyme (solid white arrowhead) with a larger expression domain of the marine allele (yellow dotted line) compared to the freshwater allele (orange dotted line) seen in both genotypes. Middle: freshwater allele still drove expression in the epithelium while marine allele had reduced or undetectable expression outside concentrated tip. The marine allele drove more robust mesenchymal expression compared to the freshwater allele. Late: marine allele drove robust expression in the mesenchyme compared to freshwater allele in mineralized tooth (dashed line). Diagram: summary of tooth epithelial and mesenchymal domains. The relative sizes of green and magenta hatched lines correspond to the approximate relative strength of expression in the epithelium. Overlapping mesenchyme domain is gray, and expanded marine mesenchyme is marked with white arrowhead. Scale bars=100 µm (B, E), 50 µm (C, F). *n*=3 fish per genotype (6 total fish), >25 teeth per fish (304 total teeth).

As a second method to compare the spatial and temporal activity of marine and freshwater enhancer alleles, we generated stable two-color transgenic lines with the two different alleles of the *Bmp6* intron 4 tooth enhancer on separate constructs: marine: mCh; freshwater: eGFP, in the opposite 5′ to 3′ direction as the endogenous locus, and marine: eGFP; freshwater: mCh, in the same 5′ to 3′ direction as the endogenous locus. First, we present qualitative assessments of enhancer activity in these lines, and then below present quantitative analyses. In adult fish, both marine and freshwater enhancers were observed to drive dynamic expression in the inner dental epithelium, more intensely at earlier stages, and diminishing as development of the tooth approaches eruption (Figure 3, A–C and Supplementary Figure S4, A–C), consistent with *Bmp6* expression detected by whole-mount ISH (Cleves *et al.* 2014; Ellis *et al.* 2016). In multiple tooth germs, a brighter focus was observed at the distal tip of the epithelium with both enhancers (Figure 3, A–C and Supplementary Figure S4, A–C), a domain resembling the localized distal epithelial expression of *Fgf10* and putative enamel knot in developing shark teeth (Rasch *et al.* 2016). This distal epithelial domain was the last epithelial region to drive reporter expression prior to cessation in the epithelium. While both enhancers were observed to drive expression in the epithelium, the freshwater allele drove seemingly more robust expression of the reporter, both in terms of intensity as well as spatial extent of the domain (Figure 3, B and C and Supplementary Figure S4, B and C).

### Doubly transgenic fish confirm reduced freshwater mesenchymal *Bmp6* enhancer activity in post-divergence fish

Reporter expression from the two alleles appeared in the mesenchyme of teeth across all stages. In pre-eruption (early and middle stage) tooth germs, condensed mesenchyme was observed to show activity of both enhancers (Figure 3, B and C and Supplementary Figure S4, B and C). In fully formed, erupted, late-stage teeth, reporter expression was observed in the mesenchymal core, extending from the tip of the core down to the base of the tooth where expression widened. Deeper mesenchyme was observed to consistently display marine but not freshwater enhancer activity. The deeper, broader, and more robust mesenchymal expression domain driven by the marine allele compared to the freshwater allele was also observed in stages of tooth development prior to eruption (Figure 3, B and C and Supplementary Figure S4, B and C).

### Reciprocal reporter/enhancer pairing in second doubly transgenic two-color line support epithelial and mesenchymal shifts in enhancer activity

To determine if the previous observations were artifacts due to factors such as transgene position effects, fluorophore used, or enhancer orientation, next we made constructs where each enhancer had an opposite enhancer orientation and drove the other fluorophore (Figure 3D). These constructs were then randomly integrated by Tol2-mediated transgenesis, representing independent genomic integrations of oppositely oriented enhancers with alternate fluorophores, simultaneously controlling for genomic position effect, enhancer orientation, and fluorophore. Using these reciprocal constructs, we again observed the epithelial and mesenchymal differences seen in the bicistronic construct and the first double transgenic line, suggesting that the QTL-associated freshwater SNPs reduce mesenchymal and expand epithelial enhancer activity (Figure 3, E and F and Supplementary Figure S4, E and F).

### Less pronounced enhancer activity differences in early fish

**Figure 4:**
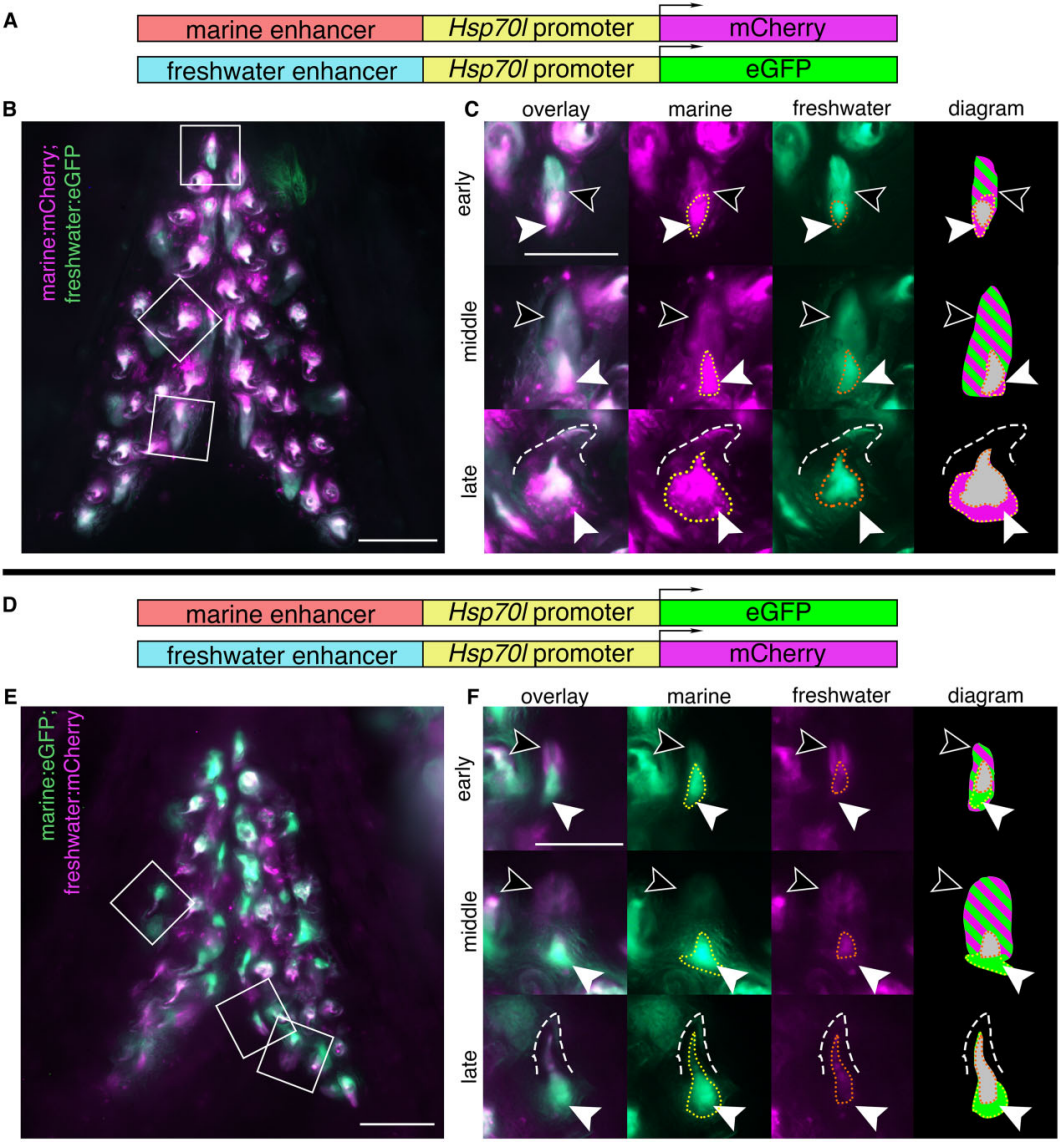
Marine and freshwater *Bmp6* enhancers drive more similar spatial patterns in younger fish. Ventral pharyngeal tooth plates from <20 mm (pre-tooth number divergence) fish doubly transgenic for two alleles of the *Bmp6* intron 4 enhancer driving two different reporter genes (A, D): the marine enhancer driving mCh with the freshwater enhancer driving eGFP (B, C) and the marine enhancer driving eGFP with the freshwater enhancer driving mCh (E, F). Bilateral ventral tooth plates (B, E) are shown next to representative teeth from the three stages (C, F): early, middle, and late highlighted by white boxes in B, E. Early: both freshwater and marine enhancer drove expression robustly in the epithelium (black arrowheads), while both enhancers drove expression in the mesenchyme (white arrowheads), the marine enhancer drove a broader domain (yellow dotted line) compared to the freshwater enhancer (orange dotted line). Middle: both enhancers continued to drive robust, apparently similar levels of expression in the epithelium (black arrows). In the mesenchyme (white arrowheads) the domain of the freshwater enhancer was reduced compared to the marine allele. Late: marine allele continued to drive a broader domain within the mesenchyme of mineralized teeth (dashed line). The relative sizes of green and magenta hatched lines correspond to the approximate relative strength of expression in the epithelium. Overlapping mesenchyme domain is gray, and expanded marine mesenchyme is marked with white arrowhead. Scale bars=100 µm (B, E), 50 µm (C, F). *n*=3 fish per genotype (6 total fish), >25 teeth per fish (249 total teeth).

Allele specific differences in the expression levels of the freshwater and marine alleles of *Bmp6*, as well as tooth number, have been shown to arise later in development (>20 mm fish length). We hypothesized that if the SNPs found within the freshwater and marine haplotypes contribute to the ASE differences, and subsequent tooth number differences, the differences in enhancer expression should be more pronounced in larger fish compared to smaller fish. Fish smaller than the tooth divergence point (∼16–18.5 mm juveniles, see *Materials and methods*) were dissected from each genotype and tooth plates were fixed and imaged (Figure 4). While the epithelial and mesenchymal expression differences observed in the older post-divergence stages were still present in both the dental epithelium and mesenchyme (Figure 4, C and F), the enhancer differences were less pronounced. In multiple early and middle stage teeth, the epithelium showed similar activity from both alleles (Figure 4, C and F), unlike the expanded freshwater epithelial domain that was observed in larger fish. Overall, the expression patterns of the two enhancers appeared more similar in pre-divergence fish, consistent with previous ASE and tooth number results (Cleves *et al.* 2014).

### Quantification of epithelial and mesenchymal expression patterns

To quantify the spatial extent of enhancer activity, we used ImageJ to quantify the reporter gene expression area of tooth mesenchyme and tooth epithelium driven by the marine and freshwater enhancers in both reciprocal two-color lines. We measured the 2D area of each transgene expression domain and then expressed the ratio of the freshwater domain area divided by the marine domain area. We first asked whether the area of enhancer expression was significantly different between the two reciprocal two-color lines (Supplementary Figure S5). In both mesenchyme (Supplementary Figure S5A) and epithelium (Supplementary Figure S5B), the expression domain areas were not significantly different between marine: mCh; freshwater: eGFP and marine: eGFP; freshwater: mCh fish.

**Figure 5:**
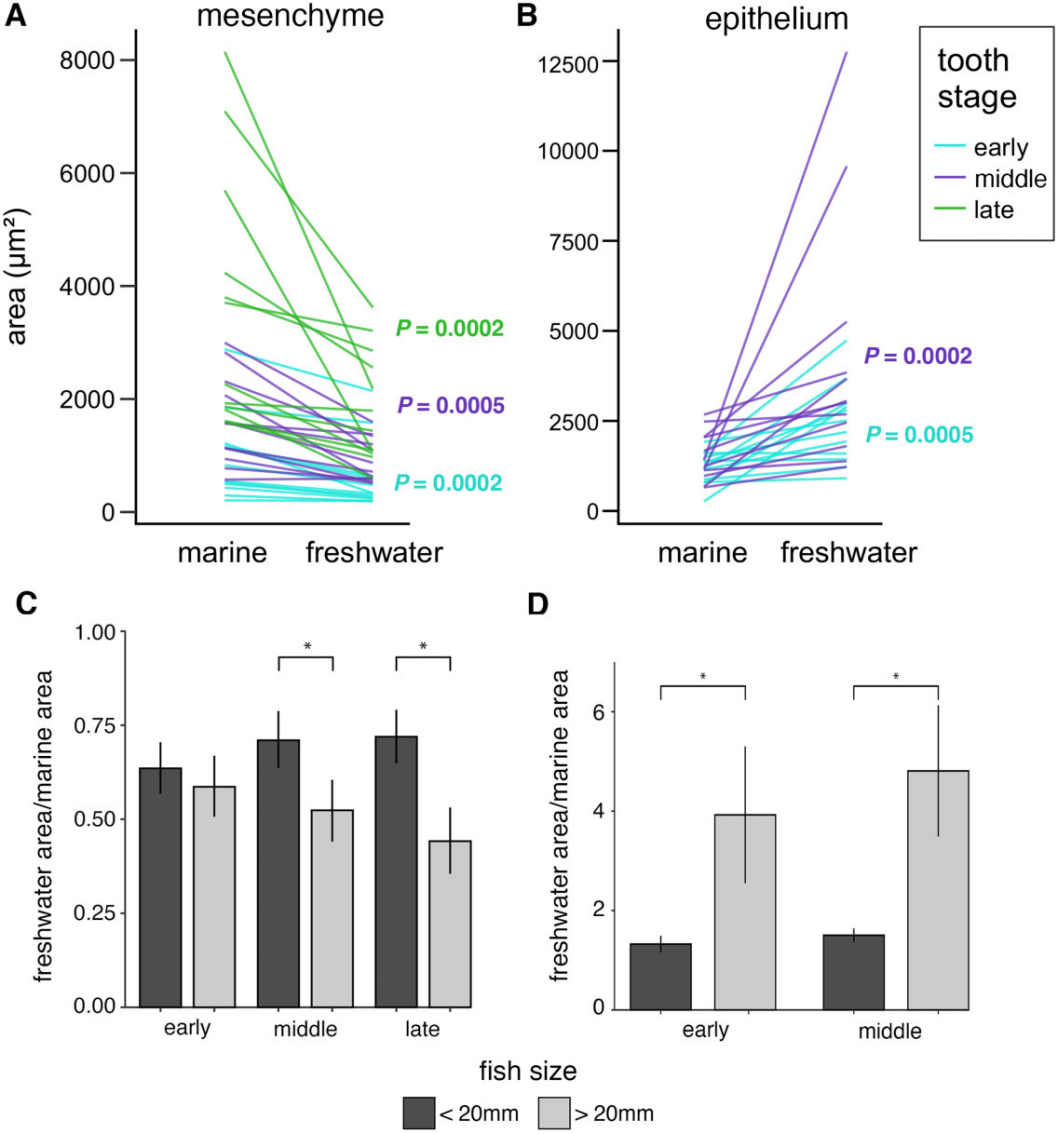
Quantification of enhancer domains supports evolved spatial and temporal shifts in enhancer activity. Area of expression domains of both the marine and freshwater enhancers in the mesenchyme (A, C) and epithelium (B, D) for one tooth in each tooth stage (early, middle, late) in three fish per genotype (marine: mCh; freshwater: eGFP, marine: eGFP; freshwater: mCh) at early and late fish stages (<20 mm, >20 mm fish total length) (total *n* = 12 fish, 36 teeth). (A) In tooth mesenchyme, the freshwater enhancer drove significantly smaller area of reporter gene expression relative to the marine allele at all three tooth stages (early, middle, late). (B) In tooth epithelium, the freshwater enhancer drove significantly larger expression domains relative to the marine allele at both early and middle tooth stages. (A–B) *P* values show Wilcoxon signed-rank one-tailed tests testing the hypotheses that the freshwater allele is reduced in the mesenchyme and expanded in the epithelium (Figures 2–4). (C) Combining genotypes but maintaining separate categories for tooth stage, the same trend was observed with the reduced mesenchymal expression domain of the freshwater allele becoming more significant in late (>20 mm) stage fish relative to early stage fish (<20 mm). Differences were significant at both middle tooth stage (*P *=* *0.046) and late tooth stage (*P *=* *0.02). (D) Combined genotypes for both early and middle stage teeth showed a significant difference when comparing <20 mm and >20 mm fish (early stage: *P *=* *0.007, middle stage: *P *=* *0.01). (C and D) *P-*values show Wilcoxon rank sum one-tailed tests testing the hypotheses that the evolved reduction in mesenchymal area (C) and expansion in epithelium (D) are greater at >20 mm fish stages than at <20 mm fish stages. Error bars show standard error of the mean and asterisks denote *P *<* *0.05. *n* = 3 fish per genotype per fish stage (12 total fish), 3 teeth per fish (36 total teeth).

We next tested the hypotheses, based upon our imaging data (Figures 2–4), that the freshwater enhancer drives a reduced mesenchymal but expanded epithelial domain relative to the marine enhancer (Figure 5). In mesenchyme, the freshwater enhancer drove a significantly smaller area than the marine enhancer (Figure 5A) at all three (early, middle, late) tooth stages. In epithelium, the freshwater enhancer drove a significantly larger area than the marine enhancer (Figure 5B) at both early and middle tooth stages.

Pooling the data of both reciprocal genotypes revealed that the relative mesenchymal area of the freshwater enhancer became significantly reduced at both middle and late tooth stages at late (>20 mm) fish stages compared to early (<20 mm) fish stages (Figure 5C). The expansion of the freshwater epithelial domain relative to the marine domain was significantly greater at late fish stages than early fish stages for both early and middle stage teeth (Figure 5D). Comparing enhancer activity between dorsal and ventral pharyngeal teeth revealed a trend toward more evolved shifts in mesenchymal expression in ventral teeth than dorsal teeth (Supplementary Figure S6A), consistent with the more pronounced phenotypic effects of the *Bmp6* QTL on ventral than dorsal tooth number (Miller *et al.* 2014).

Overall, these quantitative data support greater spatial differences of marine and freshwater enhancers at late fish stages than early fish stages, and also strongly support the conclusions that the freshwater enhancer drives a smaller mesenchymal domain and a larger epithelial domain relative to the marine enhancer.

### Pectoral and caudal fin expression differences

The *Bmp6* intron 4 enhancer was previously known to drive expression in the developing fin margins of the pectoral and median fins early in development, starting approximately 4 dpf (Cleves *et al.* 2018). In pre-hatching fish, 6 dpf, the domains of the two enhancers appear to be identical (Supplementary Figure S7A). We found that enhancer activity persists at later stages in both the pectoral and caudal fins, specifically in the intersegmental joints. The fin rays of all fins in sticklebacks consist of a series of repeated segments, made up of hemi-segments encasing a mesenchymal core like other teleosts (Haas 1962; Santamaría *et al.* 1992). In the caudal fin of both genotypes (marine: mCh; freshwater: eGFP; and marine: eGFP; freshwater: mCh), the freshwater enhancer was observed to have activity in multiple intersegmental joints, while the activity of the marine enhancer was detected in few or no joints (Supplementary Figure S7B). A similar pattern is observed in the pectoral fins (Supplementary Figure S7C). With both enhancers, more basal joints were observed to have expression, while fluorophore intensity diminished as the joints became more distal. Overall, across both fin types, the freshwater allele appeared to be active in a larger number of intersegmental joints. While more proximal intersegmental joints were more likely to have activity from both enhancers, the most proximal joint was observed to be lacking detectable reporter expression in some fin rays (Supplementary Figure S8, A and B), suggesting a dynamic cycle of initial inactivity in newly formed, distal, intersegmental joints, followed by a period of activity in most joints as they adopt a more proximal identity, and a final transition to inactivity in the proximal most joints just prior to the ultimate fusion of the basalmost segment to the next segment.

### *Bmp6* expression differences between marine and freshwater fish

**Figure 6:**
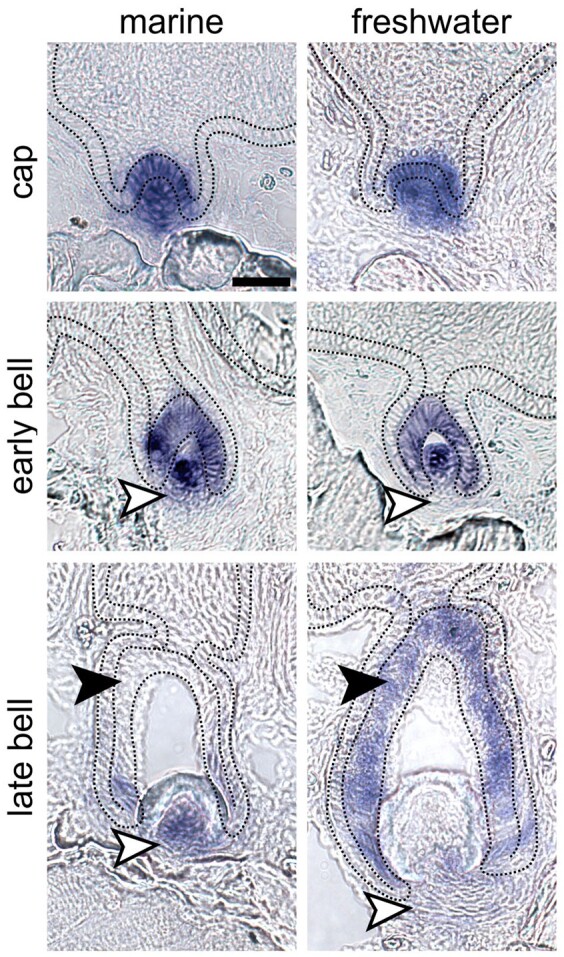
*In situ* hybridization (ISH) illustrates that *Bmp6* expression shifts mirror enhancer activity differences in marine and freshwater backgrounds. ISH of *Bmp6* expression on thin sections of marine (left column) and freshwater (right column) fish suggest that marine fish exhibit expanded mesenchymal expression at early and late bell stages (white arrowheads in middle and bottom rows, respectively), while freshwater fish exhibit relatively broader expression in the inner dental epithelium of late bell stage teeth (black arrowheads in bottom row). No expression domain differences were observed in cap stage tooth germs (top row). Marine and freshwater strains are derived from population in Rabbit Slough, AK, USA (RABS), and Paxton Lake, BC, Canada (PAXB), respectively. Black dotted lines demarcate the basalmost layer of epithelium, adjacent to the basement membrane, which includes the inner and outer dental epithelium. See Supplementary Figure S6 for DAPI counterstains and ISH images without markup. Scale bar=20 µm and applies to all panels. *n*=6 fish per population, >10 teeth per fish.

Given the consistent differences in reporter gene activity observed for the marine and freshwater enhancers, we next asked if endogenous *Bmp6* expression differed in tooth germs between marine and freshwater animals in a similar fashion. To answer this, we performed ISH on thin sections of pharyngeal tissues from marine (RABS) and freshwater (PAXB) adults (∼40 mm standard length). Marine and freshwater samples were collected, prepared, and assayed in parallel to ensure maximal comparability of the resulting data (see *Materials and methods*). While early bud and cap stage tooth germs did not show any consistent differences in gene expression, we did observe more widespread mesenchymal expression in marine tooth germs at early and late bell stages, and consistently widespread inner dental epithelial expression in freshwater epithelium relative to marine epithelium at late bell stages (Figure 6 and Supplementary Figure S9). These ISH results corroborate the reporter construct activity, suggesting that the regulation of *Bmp6* mRNA in tooth germs varies in the same direction as the variation in activity seen between the marine and freshwater *Bmp6* intron 4 enhancers.

## Discussion

### Freshwater and marine alleles of *Bmp6* tooth enhancer drive expression differences in developing teeth

Throughout the development of a tooth, multiple pathways and signals, including Bone Morphogenetic Proteins (BMPs), are involved in organ initiation and growth. Knocking out the receptor *Bmpr1a* in the dental epithelium of mice leads to arrested development of the tooth at the bud stage, demonstrating a key activating role for BMP signaling during tooth development (Andl *et al.* 2004). Overexpressing *Noggin*, a BMP antagonist, in the epithelium also results in arrest at the placode stage (Wang *et al.* 2012). In addition, in *Msx1* mutant mice, exogenous *Bmp4* can rescue tooth development (Bei *et al.* 2000). Together, these results suggest a dynamic role of *Bmp* signaling in tooth development in promoting tooth development at different stages. *Bmp6* is dynamically expressed during stickleback tooth development. Expression is detected early in the overlying inner dental epithelium as well as in the condensing underlying odontogenic mesenchyme, with a subsequent cessation of expression in the epithelium, and continuous expression in the mesenchyme of the mineralizing tooth (Cleves *et al.* 2014; Ellis *et al.* 2016). Freshwater sticklebacks homozygous for mutations in *Bmp6* have reductions in tooth number, showing *Bmp6* is required for aspects of tooth development in fish (Cleves *et al.* 2018).

**Figure 7:**
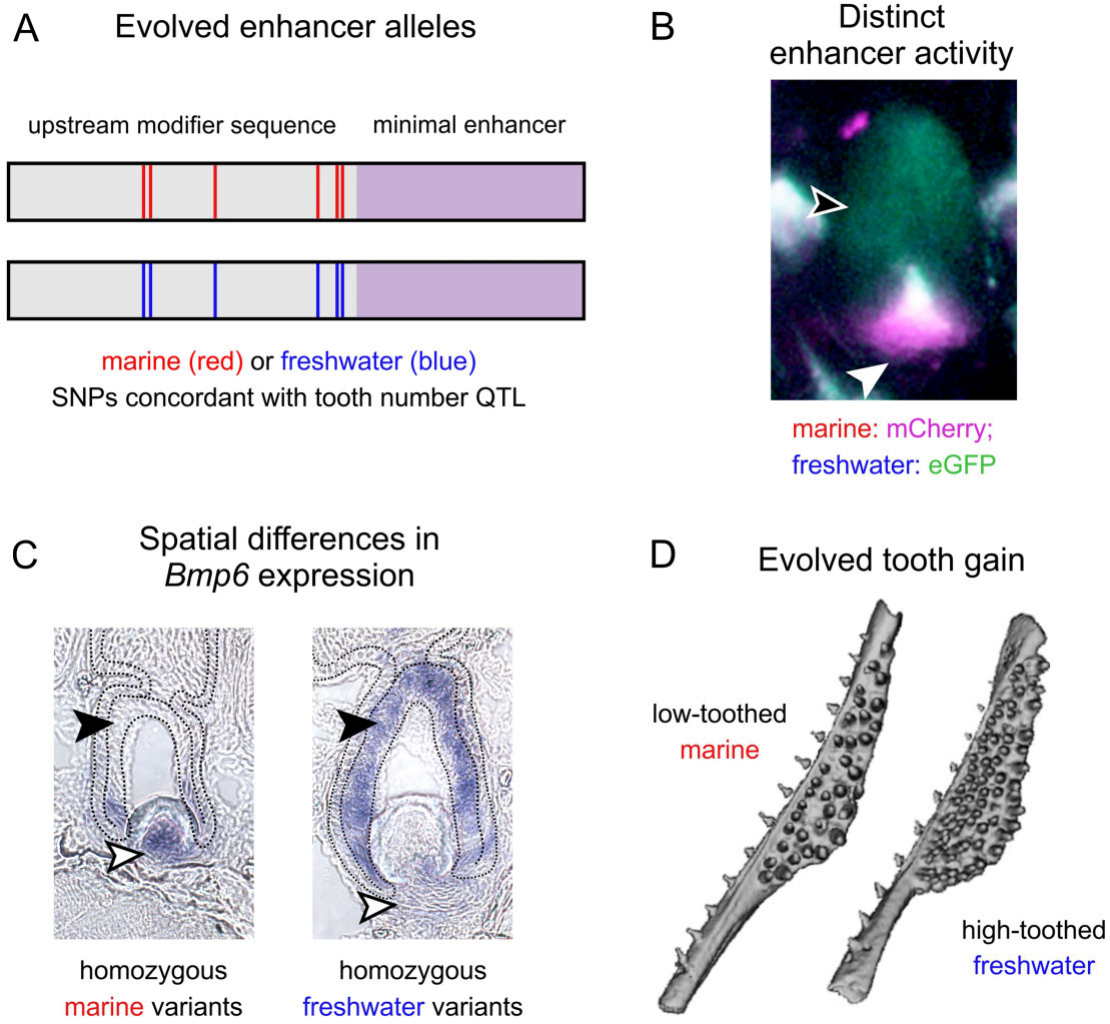
A model for the role of *Bmp6 cis*-regulatory changes in underlying evolved tooth gain in sticklebacks. (A) Quantitative trait loci (QTL) and fine mapping previously revealed variants in intron 4 of *Bmp6* that were associated with evolved tooth gain in freshwater fish (Cleves *et al.* 2014, 2018; Miller *et al.* 2014). These variants are adjacent to a previously characterized minimal enhancer (lavender) that was shown to drive expression in tooth epithelium and mesenchyme (Cleves *et al.* 2018). Six core single nucleotide polymorphisms (SNPs, depicted as red and blue lines within the modifier sequence) showed complete concordance with a large effect tooth number QTL (Cleves *et al.* 2018). (B) Marine and freshwater enhancers have different spatial activity, with the derived freshwater allele driving less mesenchymal expression, but more epithelial expression relative to the marine allele. (C) Consistent with the different enhancer activity, *Bmp6* expression by *in situ* hybridization is reduced in the mesenchyme but expanded in the epithelium in freshwater teeth relative to marine teeth. (D) We hypothesize that the enhancer alleles (A) have spatially shifted enhancer activity (B), resulting in shifts in *Bmp6* expression overall (C), and evolved tooth gain in freshwater fish (D).

A previously identified freshwater high-toothed associated haplotype within intron 4 of *Bmp6* underlies an evolved increase in tooth number. The core haplotype is defined by six polymorphic sites in the 468 bp region upstream of a minimally sufficient *Bmp6* tooth enhancer, potentially modifying enhancer activity. Three lines of evidence (the bicistronic line, and two lines of reciprocal two-color lines) support the hypothesis that the associated polymorphisms upstream of the *Bmp6* tooth enhancer result in evolved spatial shifts in enhancer activity between the marine and freshwater alleles (Figures 2–4). Both alleles drove expression in the epithelium of early developing teeth, and in dental mesenchyme throughout development, similar to the expression pattern of the adjacent minimally sufficient 511 bp tooth enhancer previously reported (Cleves *et al.* 2018) as well as the reported expression of the endogenous *Bmp6* gene during tooth development (Cleves *et al.* 2014). In all three different transgenic comparisons, we observed that the freshwater, high-toothed associated enhancer allele maintained a larger and more robust expression domain in the overlying epithelium for a longer portion of a tooth’s development compared to the marine, low-toothed associated allele. Conversely, the freshwater allele appeared to drive reporter expression in a smaller domain in the underlying mesenchyme in a large proportion of teeth. As this reduced mesenchymal and expanded epithelial activity of the freshwater enhancer relative to the marine enhancer was observed in all three transgenic lines, the enhancer differences are unlikely to be due to differences in genomic integration, enhancer orientation, or different fluorophore properties. We additionally found that marine and freshwater endogenous *Bmp6* gene expression domains differed in a manner that was consistent with the reporter gene results. Specifically, we observed larger mesenchymal domains in marine relative to freshwater fish and expanded epithelial domains in freshwater relative to marine fish, especially in late bell stage tooth germs. Together, these data support the hypothesis that the intron 4 enhancer variants associated with tooth number differences drive *Bmp6* expression differences in tooth germs of >20 mm fish, which in turn leads to evolved tooth gain in freshwater fish (Figure 7). Outstanding questions include what these deep mesenchymal cells are and whether the expanded marine mesenchymal domain might include quiescent mesenchymal cells involved in tooth replacement.

One major unanswered question remains how the spatial and temporal differences in *Bmp6* enhancer activity reported here could regulate the increases in tooth number and accelerated tooth replacement rates previously reported for freshwater fish relative to ancestral marine fish. We previously hypothesized that tooth regeneration might be regulated similarly to mammalian hair regeneration (Cleves *et al.* 2018), where BMP signaling promotes epithelial stem cell quiescence, and reducing BMP signaling in mouse skin accelerates hair regeneration (Kandyba *et al.* 2013). Multiple lines of gene expression data have supported this hypothesis (Cleves *et al.* 2018; Hart *et al.* 2018; Square *et al.* 2021). However, several other compelling, and not necessarily mutually exclusive, alternative hypotheses include (1) tooth regeneration might be regulated by coordinated cyclic waves involving BMP signaling, similar to the cyclic nature of hair regeneration previously reported in mice (Plikus *et al.* 2008), and (2) tooth regeneration might be regulated by a reaction-diffusion system in which BMPs act as inhibitors (Kondo and Miura 2010), similar to a proposal previously made for shark denticle formation (Cooper *et al.* 2018). This first alternative hypothesis of cyclic waves coordinating tooth replacement is reminiscent of the decades old Zahnreihen theory that posits that tooth replacement in polyphyodonts is coordinated across the dentition, usually occurring at alternating tooth positions (Edmund 1960). For all these hypotheses, the increased epithelial and/or the decreased mesenchymal enhancer activity of *Bmp6* could be the causative change leading to evolved tooth gain. Future experiments will continue to test these hypotheses, test whether the epithelial and mesenchymal *Bmp6* enhancer shifts are regulated by the same or different mutations, and ultimately determine how the intronic *Bmp6* enhancer haplotype identified by our previous genetic mapping studies regulates increases in tooth number.

Previous ASE experiments demonstrated a 1.4-fold reduction in the freshwater *Bmp6* allele compared to the marine in F_1_ hybrid adult tooth tissue that included the entire ventral pharyngeal jaw, and thus both tooth epithelial and mesenchymal cells (Cleves *et al.* 2014). The mesenchymal biases in reporter expression are consistent with the ASE result, with more robust mesenchymal expression driven by the marine allele compared to the freshwater allele potentially responsible for the higher expression of the marine allele in the ASE experiments. In contrast, the expanded freshwater epithelial enhancer domain is not consistent with the overall ASE result in which freshwater alleles had *cis*-regulatory downregulation relative to marine alleles. Since the reduced mesenchymal domain in the freshwater enhancer relative to the marine enhancer was the most striking qualitative difference, it is possible that the epithelial bias, with a stronger signal driven by the freshwater enhancer, is quantitatively canceled out by the bias in the mesenchyme, explaining the overall reduction of freshwater *Bmp6* expression compared to marine *Bmp6* expression in F_1_ hybrids.

The enhancer expression differences were significantly greater in larger, post-tooth number divergence fish compared to smaller, pre-tooth number divergence fish. While the mesenchyme appeared to have a somewhat reduced difference of expression between the two alleles, the epithelium demonstrated less pronounced differences in activity between the alleles in pre-divergence fish. These observations are consistent with ASE results and the divergence in tooth number in marine and freshwater fish. While the mesenchymal difference was still observable early, it is possible that there are other regulatory regions which act as repressors for the marine *Bmp6* allele or enhancers for the freshwater *Bmp6* allele early in development and so mask the mesenchymal bias of the intron 4 enhancer. For example, we previously reported a 5′ *Bmp6* tooth enhancer that also contributes to the overall pattern of *Bmp6* expression in developing teeth (Erickson *et al.* 2015).

Future experiments to measure ASE in isolated tissues, with epithelium and mesenchyme separated, could test whether opposing quantitative differences are present in dental epithelium *vs* mesenchyme, as the new data presented here suggest. A quantitative method could be used to further test the hypothesis that the two enhancers drive differing levels of expression, such as pyrosequencing (Wittkopp 2012) with the two enhancers both driving identical fluorophores, with a single synonymous mutation distinguishing the two. Alternatively, single-cell RNA-seq in the dental epithelium and mesenchyme, tracking the respective reporters of each enhancer, could determine if there are quantifiable expression differences between the two enhancers.

### QTL-associated sequence difference in alleles may underlie expression domain differences and evolved tooth gain

There are 14 point mutations and three indels distinguishing a low-toothed marine (Little Campbell) allele from the high-toothed Paxton Lake allele of the intron 4 enhancer in our reporter constructs. Previous experiments identified ten SNPs that co-occur consistently with the presence or absence of a tooth number QTL and of these ten, the core six are present in the enhancer reporter constructs tested here (Cleves *et al.* 2018). From our results, we are unable to distinguish whether these six polymorphisms contribute to the expression differences we observed. While it is possible that the three indels or the eight non-QTL-associated SNPs may contribute, it is an attractive and parsimonious hypothesis that the same SNPs that co-occur with the tooth QTL are also responsible for the reporter expression differences, and the previously described ASE results. Of the six QTL-associated SNPs tested here, of special interest is the second QTL-associated SNP, which in the freshwater allele, creates a predicted NFATc1 binding site (Cleves *et al.* 2018). NFATc1 was shown to be required for balancing of quiescent and actively dividing stem cells in hair follicles (Horsley *et al.* 2008) which share homology with teeth (Pispa and Thesleff 2003; Biggs and Mikkola 2014; Ahn 2015), and so a difference in NFATc1 binding may potentially play a role in the *Bmp6* ASE and enhancer activity differences observed previously and here. Supporting this hypothesis, *Nfatc1b* expression was recently shown to be present in stickleback tooth germs and functional tooth mesenchyme (Square *et al.* 2021).

To better determine which polymorphisms may underlie the expression differences we observed, hybrid enhancers can be made. For example, if the creation of an NFATc1 binding site is at least partially responsible for the observed differences, a marine allele with the SNP converted to the freshwater identity, from a “C” to a “T,” may recapitulate the freshwater enhancer expression patterns. By creating and testing hybrid enhancers, future experiments could test which enhancer polymorphisms alone and in combination contribute to the expression differences reported here.

### Fin expression differences

In addition to the reporter expression differences driven by the two enhancers during tooth development, we observed distinct expression patterns in the pectoral and caudal fins. It was previously known that the minimal 511 base pair enhancer drove expression in the margins of early pectoral and median fins, but expression in adult fins had not been described. BMP signaling plays a role in fin regeneration, with BMP inhibition reducing osteoblast differentiation in new cells arising at the leading edge of the regenerating fin (Stewart *et al.* 2014). During zebrafish fin regeneration, *bmp2b*, *bmp4*, and *bmp6* are expressed, and are thought to be important (Laforest *et al.* 1998; Murciano *et al.* 2002; Quint *et al.* 2002; Smith *et al.* 2006). While both alleles of the *Bmp6* enhancer drive expression in the pectoral and caudal fins of sticklebacks, the differing enhancer activities may result in developmental differences, through osteoblast function in the developing lepidotrichia and intersegmental joints, possibly leading to different fin morphologies and/or regenerative abilities. Differences in expression of *bmp2* have been observed in the regeneration of different rays of the caudal fin in cichlids (Ahi *et al.* 2017), as well as the expression of the gene *msxb*, which is downstream of *bmp* signaling in the regenerating zebrafish fin (Smith *et al.* 2006).

Multiple studies have identified habitat specific differences in fin morphology (Taylor and McPhail 1986; Kristjánsson *et al.* 2005; Hendry *et al.* 2011). As the two enhancers are derived from populations with two distinct ecotypes, a benthic freshwater population, and a highly mobile anadromous population, it is possible that this enhancer may influence pectoral and caudal fin size and shape in an adaptive manner. Characterization of fin morphology using fish from either a population in which the high-toothed and low-toothed associated haplotypes are segregating, or those from a control cross in which both alleles were present in the founding, could test whether there is a fin morphology difference associated with the different alleles.

### Bicistronic constructs and the use of genetic insulators

Simultaneous comparison of two enhancer alleles in a single organism via a bicistronic construct is an attractive means to compare molecularly divergent enhancers (*e.g.*, pairs of enhancers that contain sequence variation across populations to determine if there are population-specific differences in enhancer activity). Previous work in zebrafish utilized genetic insulators as part of an enhancer trap as well as with two different tissue-specific promoters and demonstrated the effectiveness of the technique (Bessa *et al.* 2009; Shimizu and Shimizu 2013).

Here, we used a bicistronic construct with a *Bmp6* enhancer and a *Col2a1a* enhancer driving different fluorophores in mosaically transgenic F_0_ fish to test whether the activities of two enhancers could be insulated from each other. Within the same F_0_ individual, some domains demonstrated a high degree of insulator effectiveness while others did not. There are at least two possible explanations: (1) the insulated *vs* non-insulated regions represent distinct and mosaic integration events, with the insulator effectiveness determined by the integration site in a particular subpopulation of cells, or (2) the same integration event can differ in insulator behavior stochastically or based on some context that differs from an insulated expression domain to an un-insulated domain. Regardless, examining enhancer activity in stable lines will still provide a more complete picture of the role of the regulatory element and has advantages over mosaic F_0_ analyses.

Genetic insulators have been reported to limit enhancer activity across the insulator boundary (Bessa *et al.* 2009; Shimizu and Shimizu 2013) as well as protect against position effects (Chung *et al.* 1993), while other experiments show a lack of protection (Grajevskaja *et al.* 2013). The insulator used here, from the 5′ end of the mouse tyrosinase locus, was reported to bind CTCF (CCCTC-binding factor), like the β-globin 5′-HS4 insulator from chicken, and is reported to prevent influences from nearby chromatin state and gene activity, the hallmarks of genetic insulators (Montoliu *et al.* 1996; Giraldo *et al.* 2003; Molto *et al.* 2009). As there are conflicting reports of the use of insulators to fully shield from nearby chromatin states and position effects, the combined use of a landing pad locus could help to further reduce these effects (Roberts *et al.* 2014). We recommend a multipronged approach utilizing multiple transgenic lines (*e.g.*, either bicistronic constructs or multiple independent reciprocal two-color lines where each enhancer drives a different fluorophore in the same animal). Similar methods in doubly transgenic animals should allow future dissection of spatial differences in enhancer alleles, with the two methods acting as means of independent verification.

Changes in *cis*-regulation of developmental genes can be an important driver of morphological evolution, as well as human disease. The impact of mutations in *cis*-regulatory regions can be difficult to predict, and if the effect is subtle or slight, also to detect. The use of two enhancers in the same individual, either as parts of two independent transgenes or within a single bicistronic construct, can both control for the trans-environment and make even slight differences in expression activity apparent due to simultaneous imaging of reporter genes driven by both enhancers. Such an approach allows for directly comparing molecularly divergent regulatory elements, potentially identifying causal polymorphisms with important developmental and evolutionary implications.

## Data availability

Strains and plasmids are available upon request. The authors affirm that all data necessary for confirming the conclusions of the article are present within the article, figures, and tables.

Supplementary material is available at *GENETICS* online.

## Supplementary Material

iyab151_Supplementary_Data
