# Supplementary material for: Evolved Bmp6 enhancer alleles drive spatial shifts in gene expression during tooth development in sticklebacks
Source: Genetics. 2021 Sep 23;219(4):iyab151. doi: 10.1093/genetics/iyab151 (PMC8664583; doi:10.1093/genetics/iyab151)
Supplement: iyab151_Supplementary_Data [file iyab151_supplementary_data.pdf]

Supplemental Materials for:

Evolved *Bmp6* enhancer alleles drive spatial shifts in gene expression during tooth development in sticklebacks

Mark D. Stepaniak, Tyler A. Square, and Craig T. Miller

Department of Molecular and Cell Biology, University of California-Berkeley, Berkeley CA, 94720, USA

Figures S1-S9
Table S1-S4
Supplemental Methods
Supplemental Results

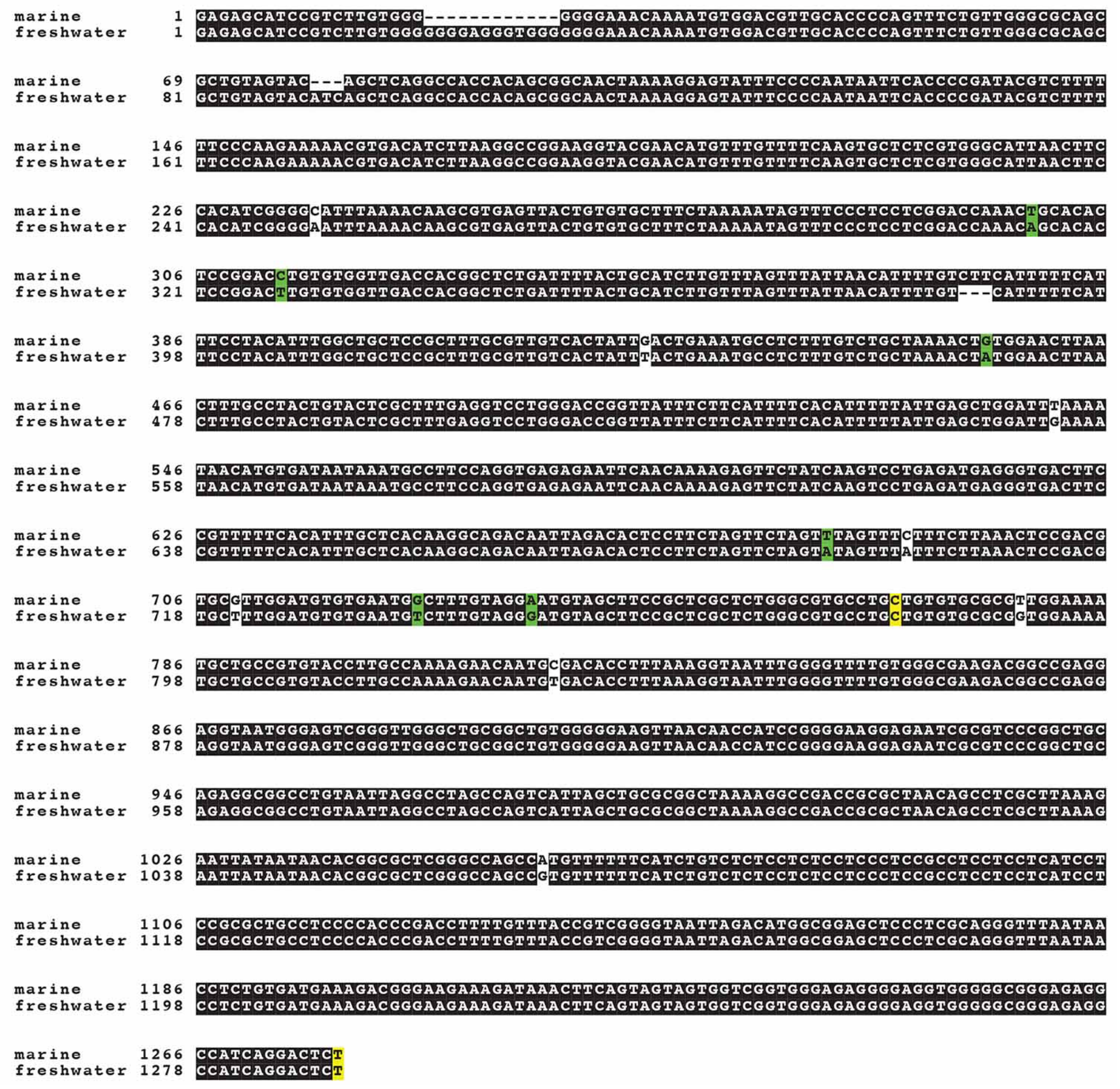

Figure S1. Sequence alignment of marine and freshwater alleles of *Bmp6* tooth enhancer
Six core single nucleotide polymorphisms (green) concordant with the presence or absence of a large effect tooth number QTL lie upstream of a ~511 bp minimal *Bmp6* tooth enhancer (start and end in yellow). Other polymorphisms (white) are not concordant with the presence or absence of the tooth QTL (Cleves *et al.* 2018).

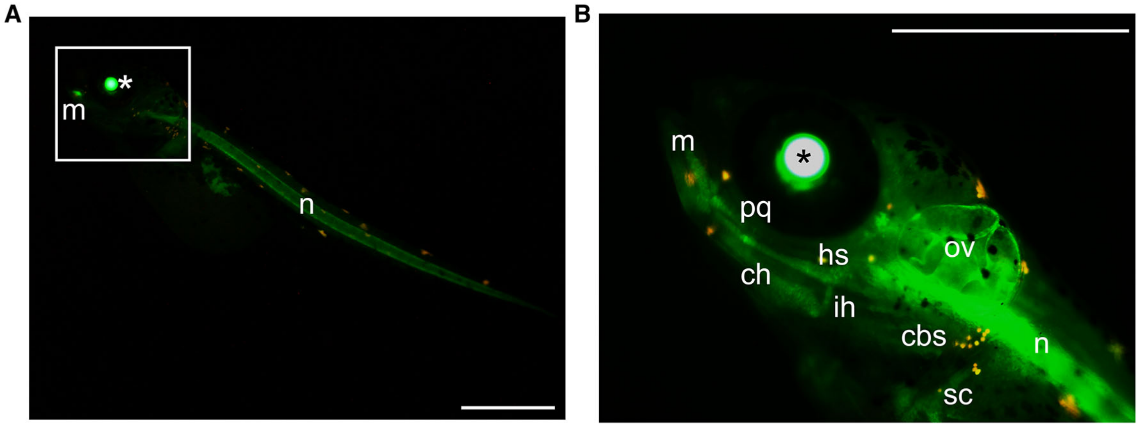

Figure S2. A *Col2a1* enhancer drives reporter expression in craniofacial cartilage and notochord in developing stickleback embryos. (**A**) In a ten day post-fertilization embryo, reporter expression was observed in the notochord (n) and Meckel's cartilage (m) and (**B**) all other craniofacial cartilages including the palatoquadrate (pq), ceratohyal (ch), interhyal (ih), hyosympletic (hs), and ceratobranchials (cbs). Expression was also seen in the scapulocoracoid (sc), and otic vesicle (ov). The lens positive control domain driven by the *Hsp70l* promoter is marked with an asterisk. Scale bars = 500µm. n > 10 fish.

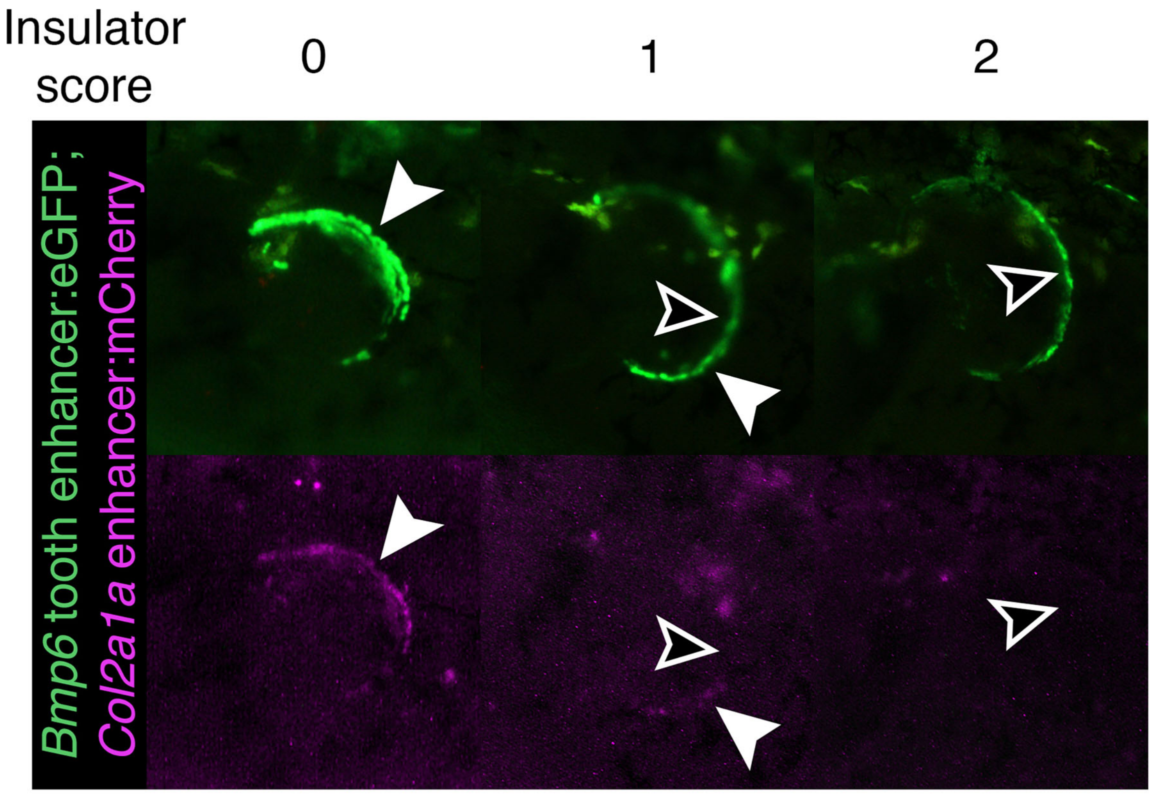

Figure S3. Insulator scoring scale in F_0 injected fish
Examples of each insulator efficiency score. Fish were injected with a bicistronic construct (*Col2a1a* enhancer driving mCherry, *Bmp6* intron 4 enhancer driving eGFP, separated by the mouse tyrosinase insulator) and domains were scored for insulator activity. A score of 0 was assigned for a domain in which both fluorophores were present (white arrowhead) throughout the same extent of the domain, indicating a lack of insulation. A score of 1 was assigned for a domain in which fluorophores were co-expressed in only a portion of the observed domain (white arrowhead), while there were also regions in which only a single fluorophore was observed (black arrowhead), indicating partial insulation activity. A score of 2 was assigned for a domain in which the predicted fluorophore was the only signal present (black arrowhead). White arrow heads indicate regions in which both fluorophores were observed, black arrowheads indicate regions in which only the predicted reporter was observed. n = 92 embryos.

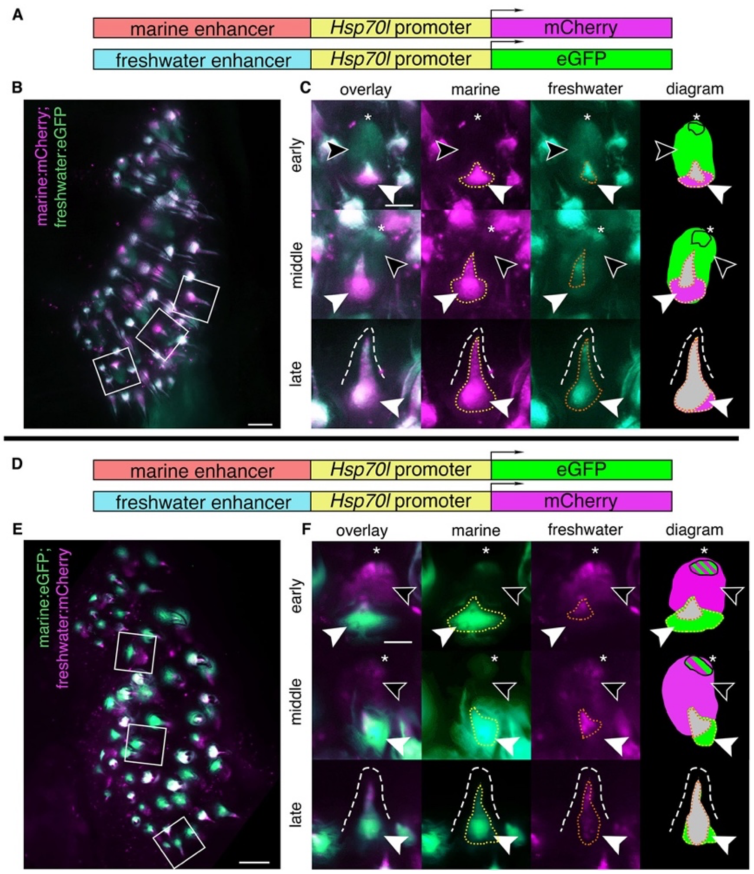

Figure S4. Marine and freshwater *Bmp6* enhancers drive different spatial patterns in dorsal pharyngeal teeth.

Dorsal pharyngeal tooth plates from fish doubly transgenic for two alleles of the *Bmp6* intron 4 enhancer driving two different reporter genes (**A,D**): the marine enhancer driving mCherry with the freshwater enhancer driving eGFP (**B,C**) and the marine enhancer driving eGFP with the freshwater enhancing driving mCherry (**E,F**). Unilateral dorsal pharyngeal tooth plates (**B,E**) are shown, next to representative teeth from three stages (**C,F**): early, middle, and late highlighted by white boxes in **B,E**. (**C,F**) Early: freshwater enhancer drove expression in the epithelium (black arrowheads), with concentrated expression in the tip (asterisk), while the marine enhancer did not reliably drive expression in the epithelium, but was observed in the distal tip (**F**) in some instances. Both enhancers also drove expression in the mesenchyme (solid white arrowhead) with a larger expression domain of the marine allele (yellow dotted line) compared to the freshwater allele (orange dotted line). Middle: freshwater allele still drove expression in the epithelium while the marine allele was restricted to the distal tip. The marine allele drove more robust mesenchymal expression compared to the freshwater allele. Late: marine allele drives robust expression in the mesenchyme compared to freshwater allele in mineralized tooth (dashed line). Diagram: summary of tooth epithelial and mesenchymal domains. The relative sizes of green and magenta hatched lines correspond to the approximate relative strength of expression in the epithelium. Overlapping mesenchyme domain is gray, and expanded marine mesenchyme is marked with white arrowhead. Scale bars = 100µm (**B**,**E**), 50µm (**C**, **F**). n = 3 fish per genotype (6 total fish), >25 teeth per fish (298 total teeth).

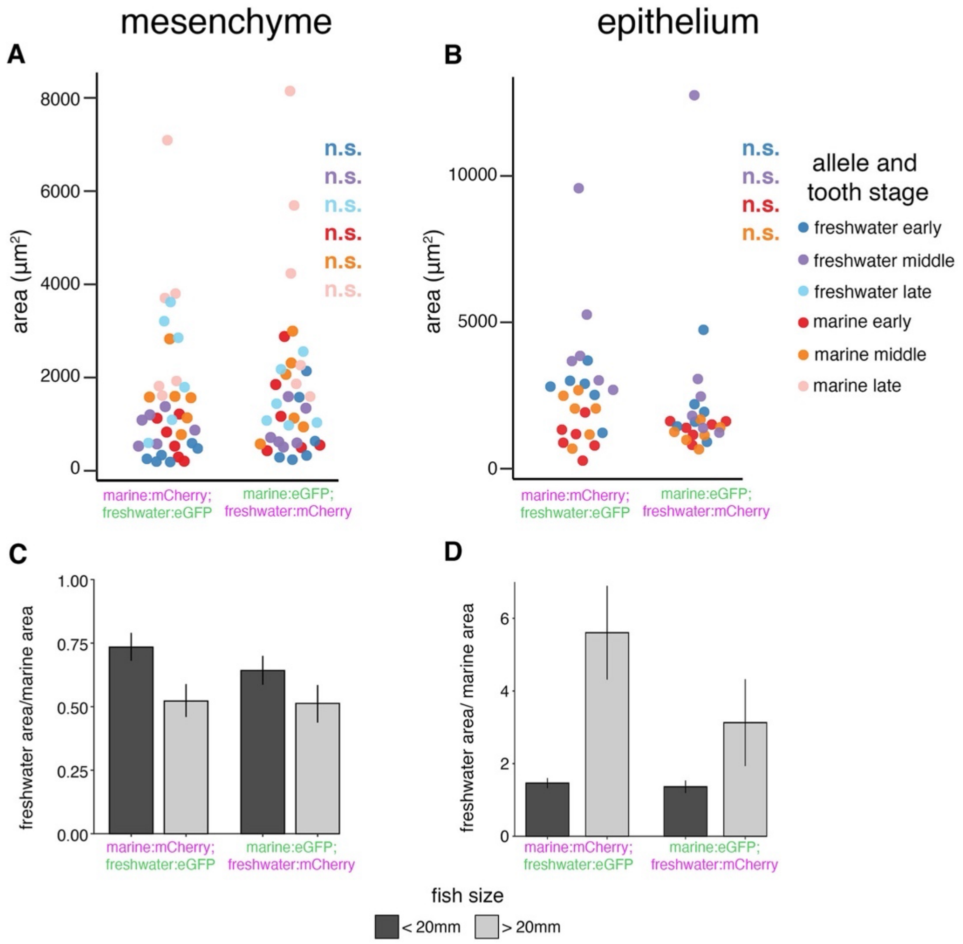

Figure S5.

(**A,B**) Area of reporter gene expression in the mesenchyme (**A**) and epithelium (**B**) of teeth at three different tooth stages (early, middle, late). In both mesenchyme and epithelium, no significant differences were observed between any tooth stage for the marine:mCherry; freshwater:eGFP and marine:eGFP; freshwater:mCherry genotypes ("n.s." = not significant, $P > 0.05$, Wilcoxon rank sum two-tailed test)(**C-D**) Pooling all stages in a genotype also displayed the same trend with greater freshwater reductions in mesenchyme (**C**) and expansions in the epithelium (**D**) at late (> 20mm) fish stage than early (< 20mm) fish in both reciprocal genotypes. In C and D, three teeth per fish were included, so no statistical tests were performed as samples are not all independent. n = 3 fish per genotype per fish stage (12 total fish), 3 teeth per fish (36 total teeth).

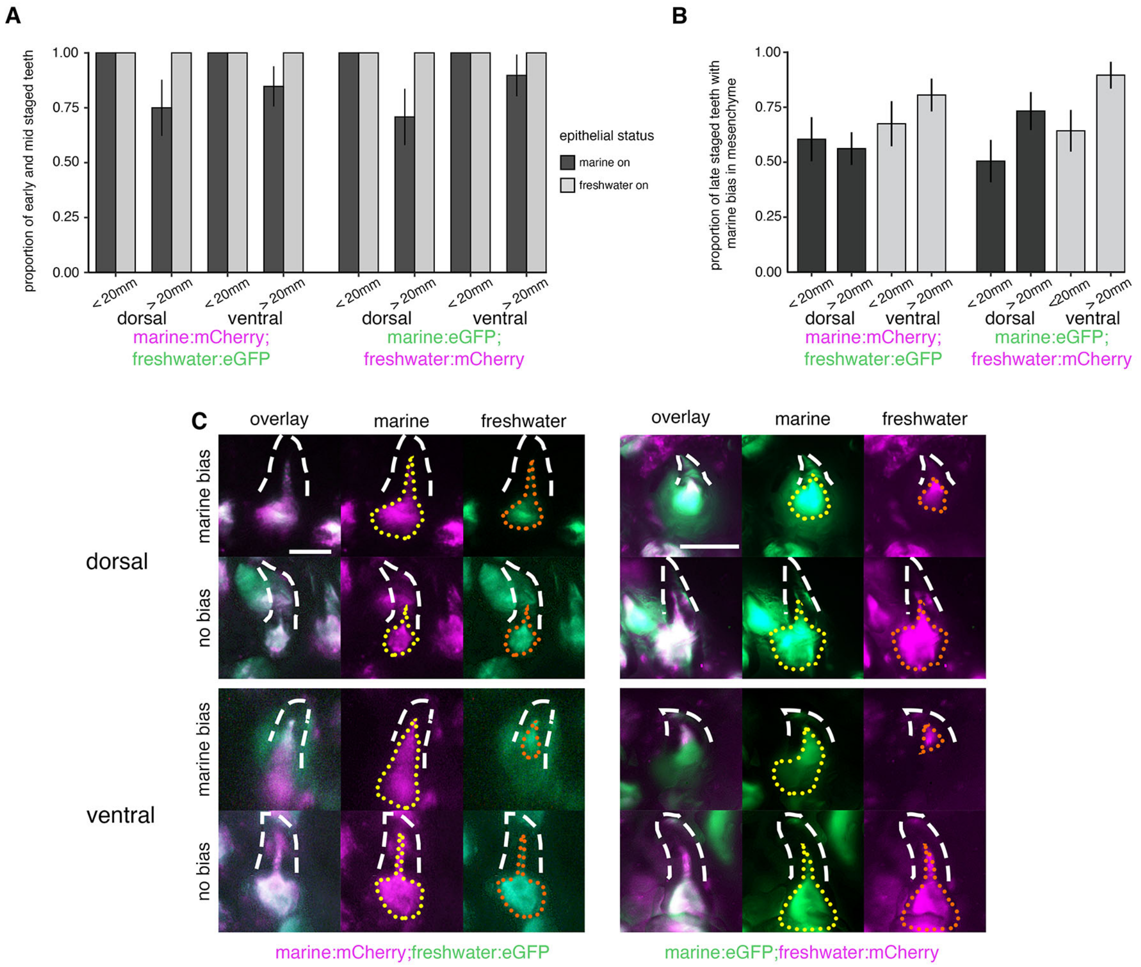

Figure S6. Differences in enhancer activity vary based on dorsal vs. ventral tooth field, fish total length, and epithelial vs. mesenchymal domain. (**A**) In < 20mm total length (pre-tooth number divergence) fish, the marine and freshwater alleles were expressed in the epithelium of all developing tooth germs regardless of genotype, while in > 20 mm total length (post-tooth number divergence) fish epithelial expression differences were consistent across tooth plates and genotypes. The freshwater allele consistently drove expression in all tooth germs scored, while the marine allele did not. Error bars show 95% C.I.s (**B**) The proportion of erupted teeth that demonstrated an observed mesenchymal bias of an expanded marine enhancer domain differed across dorsal and ventral tooth plates (dorsal and ventral, respectively), with more bias ventrally than dorsally. (**C**) Examples of erupted teeth (white dashed lines) from both dorsal and ventral tooth plates that were scored as either having a marine bias in the mesenchyme [if the freshwater enhancer mesenchymal domain (orange dotted line) was more restricted compared to the marine enhancer domain (yellow dotted line)], or no bias if the freshwater enhancer mesenchymal domain was equivalent to the marine enhancer domain. Scale bars = 50µm (**C**). n = 3 fish per genotype per fish size class (12 total fish), >50 teeth per fish (1108 total teeth, see Table S3).

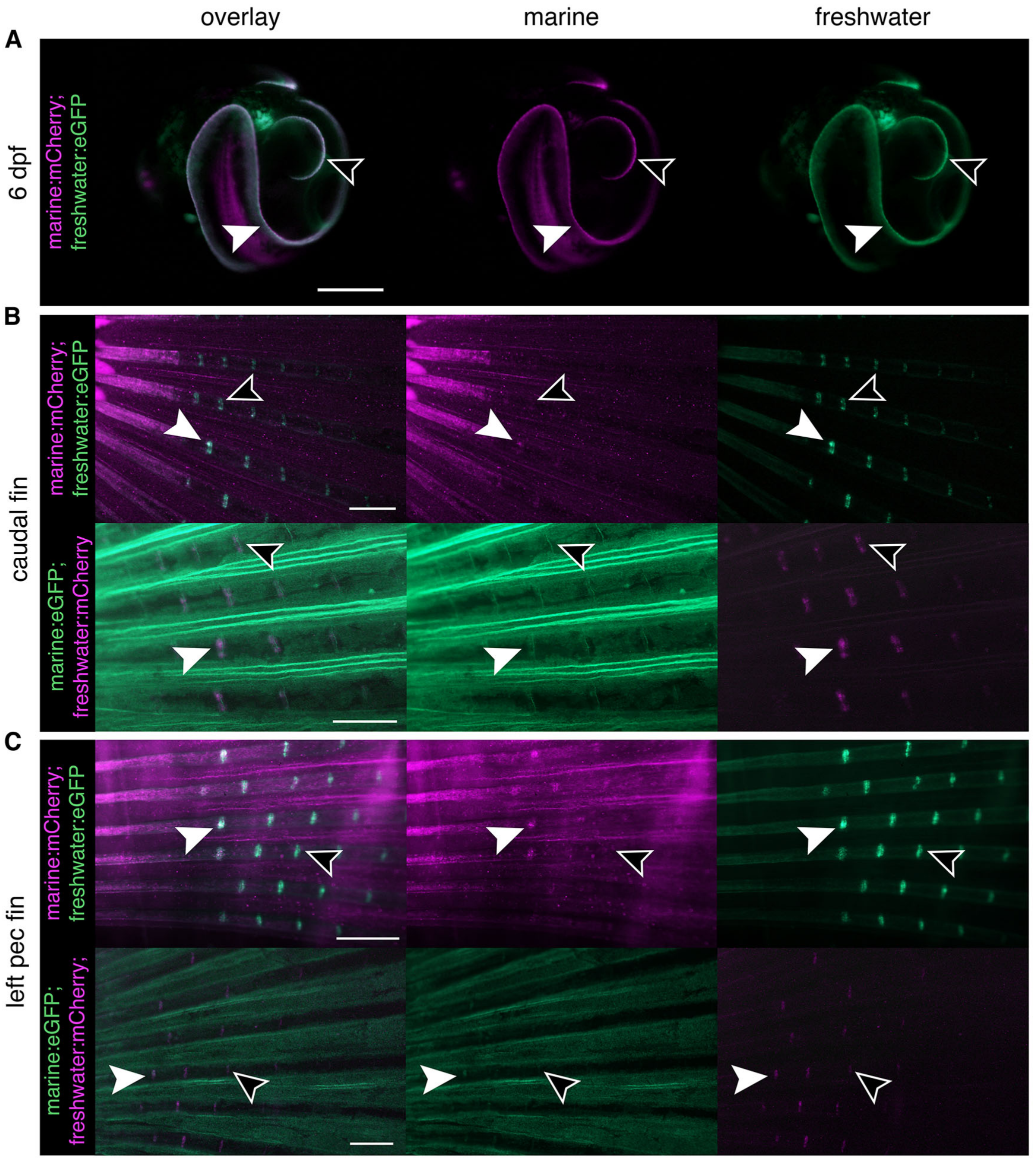

Figure S7. Freshwater allele drives expression in more intersegmental joints of both pectoral and caudal fins compared to the marine allele.
(**A**) In young, pre-hatching fish (6 dpf) the marine and freshwater enhancers drive expression in identical patterns in the developing fin margins of the pectoral fins (solid white arrowhead) and median fin (black arrowhead). (**B**) In adult caudal fins the more basal intersegmental joints were observed to have activity from both the marine and freshwater alleles (solid white arrowhead) while more distal joints were observed to only have freshwater enhancer activity (black arrowhead). The pattern was observed across both enhancer/reporter pairings. (**C**) Left pectoral fins from adults were observed to have activity from both enhancers in more basal intersegmental joints (solid white arrowheads) while only the freshwater allele was observed to have activity in more distal joints (empty arrowheads). Scale bars = 0.5 mm. n > 6 fish per genotype and >3 fish per stage.

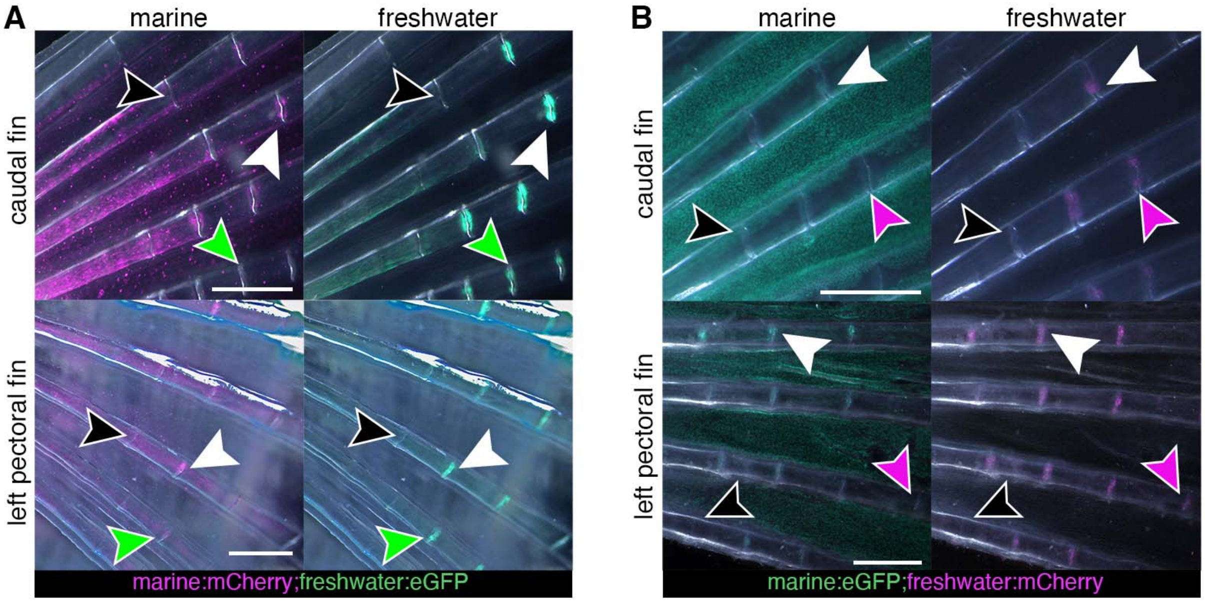

Figure S8. Fin expression patterns of both alleles change over developmental time.
(**A)** Caudal and pectoral fins with the freshwater enhancer driving eGFP and marine enhancer driving mCherry. Only the freshwater enhancer is active in more distal joints (green arrowhead) while in more basal joints both enhancers are active (solid white arrowhead). No enhancer activity was observed in the most basal joints (black arrowhead). (**B**) Caudal and pectoral fins with the freshwater enhancer driving mCherry and marine enhancer driving eGFP. Similar to (**A**), the freshwater allele is active in more distal joints than the marine allele (purple arrowhead), more basal joints exhibit activity from both enhancers (solid white arrowhead). In the most basal joints, activity from either enhancer was not observed (black arrowhead). Scale bars = 0.5mm. n > 6 fish per genotype.

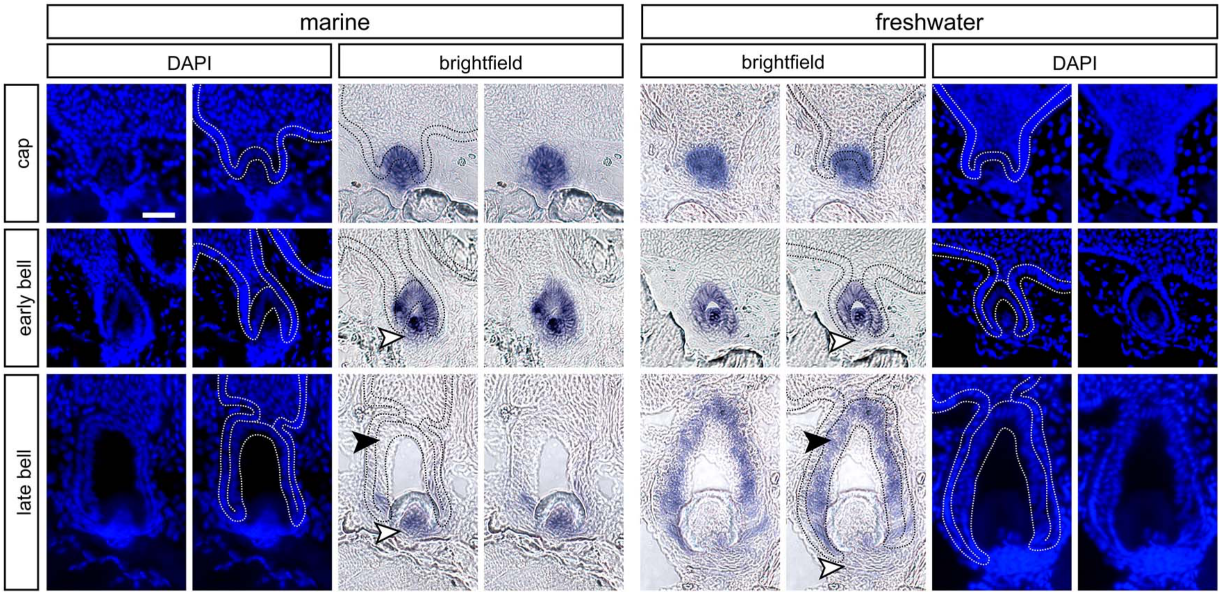

Figure S9. DAPI counterstain distinguishes between epithelial and mesenchymal tissues on thin sections. Inner four columns show brightfield *in situ* hybridization (ISH) images for *Bmp6* expression on marine (left) and freshwater (right) backgrounds, innermost columns with no annotations, adjacent to the same images with annotations (as presented in Figure 7). The outermost four columns show DAPI counterstains of the same sections, again shown both with and without annotations. The first row shows a cap stage tooth, the second row shows an early bell stage tooth, and the third row shows a late bell stage tooth. All dotted lines (black in brightfield images, white in DAPI images) demarcate the basalmost layer of epithelium in the tooth field, which is contiguous with the inner and outer dental epithelia of tooth germs. Regions where differences in expression were detected are marked with arrowheads: white arrowheads mark expanded mesenchymal expression in marine relative to freshwater, while black arrowheads mark expanded epithelial expression in freshwater relative to marine (as shown in Figure 7). Scale bar = 20 μm and applies to all panels. n = 6 fish per population, >10 teeth per fish.

Table S1. Insulator scores for bicistronic *Col2a1a:*mCh;*Bmp6* tooth enhancer:eGFP transgene

Domain	"0"- apparent no insulation	"1" – partial insulation observed	"2"- apparent complete insulation	Total fluorescence positive domains
Left pec fin	24	13	28	65
Right pec fin	28	14	21	63
Median fin	34	23	29	86
Notochord	9	1	3	13
Total	95	51	81	227

For each reporter positive domain in F_0 fish with *Col2a1a:*mCh;*Bmp6* tooth enhancer:eGFP transgene, a score of 0-2 was given for observed non, partial, or complete insulation. See Figure S3 for examples of 0-2 scores.

Table S2. Insulator scores for bicistronic *Col2a1a:*eGFP;*Bmp6* tooth enhancer:mCh transgene

Domain	"0"- apparent no insulation	"1" – partial insulation observed	"2"- apparent complete insulation	Total fluorescence positive domains
Left pec fin	12	2	4	18
Right pec fin	6	4	3	13
Median fin	15	4	3	22
Notochord	5	0	5	10
Total	38	10	15	63

For each reporter positive domain in F_0 fish with *Col2a1a:*mCh;*Bmp6* tooth enhancer:eGFP transgene, a score of 0-2 was given for observed non, partial, or complete insulation. See Figure S3 for examples of 0-2 scores.

Table S3. Epithelial expression of enhancer by tooth plate, tooth stage, and genotype.

tooth plate	time point	stage	freshwater positive (N/%)	marine positive (N/%)	total teeth in stage	genotype
DTP	pre-divergence	early	20/100%	20/100%	20	freshwater:eGFP;marine:mCherry
DTP	post-divergence	early	29/100%	24/82.8%	29	freshwater:eGFP;marine:mCherry
DTP	pre-divergence	mid	16/100%	16/100%	16	freshwater:eGFP;marine:mCherry
DTP	post-divergence	mid	15/100%	9/60.0%	15	freshwater:eGFP;marine:mCherry
VTP	pre-divergence	early	19/100%	19/100%	19	freshwater:eGFP;marine:mCherry
VTP	post-divergence	early	23/100%	20/87.0%	23	freshwater:eGFP;marine:mCherry
VTP	pre-divergence	mid	22/100%	22/100%	22	freshwater:eGFP;marine:mCherry
VTP	post-divergence	mid	36/100%	30/83.3%	36	freshwater:eGFP;marine:mCherry
DTP	pre-divergence	early	13/100%	13/100%	13	freshwater:mCherry;marine:eGFP
DTP	post-divergence	early	24/100%	18/75.0%	24	freshwater:mCherry;marine:eGFP
DTP	pre-divergence	mid	16/100%	16/100%	16	freshwater:mCherry;marine:eGFP
DTP	post-divergence	mid	24/100%	16/66.7%	24	freshwater:mCherry;marine:eGFP
VTP	pre-divergence	early	16/100%	16/100%	16	freshwater:mCherry;marine:eGFP
VTP	post-divergence	early	23/100%	21/91.3%	23	freshwater:mCherry;marine:eGFP
VTP	pre-divergence	mid	13/100%	13/100%	13	freshwater:mCherry;marine:eGFP
VTP	post-divergence	mid	16/100%	14/87.5%	16	freshwater:mCherry;marine:eGFP

For each tooth field (dorsal or ventral pharyngeal tooth plate, DTP or VTP), stage (pre-divergence = <20 mm fish length, post-divergence = >20 mm fish length, tooth stage [early or middle (mid), see Methods], the number (N), percentage (%) of detected epithelial expression are listed, along with total number of teeth and genotype of transgene.

Table S4. Mesenchymal bias of enhancer expression by tooth plate, tooth stage, and genotype.

tooth plate	time point	stage	unbiased mesenchymal expression (N/%)	biased mesenchymal expression (N/%)	Total teeth in stage	genotype
DTP	pre-divergence	early	3/15%	17/85%	20	freshwater:eGFP;marine:mCherry
DTP	post-divergence	early	1/3.4%	28/96.6%	29	freshwater:eGFP;marine:mCherry
DTP	pre-divergence	mid	2/12.5%	14/87.5%	16	freshwater:eGFP;marine:mCherry
DTP	post-divergence	mid	0/0%	15/100%	15	freshwater:eGFP;marine:mCherry
DTP	pre-divergence	late	36/39.6%	55/60.4%	91	freshwater:eGFP;marine:mCherry
DTP	post-divergence	late	46/43.8%	59/56.2%	105	freshwater:eGFP;marine:mCherry
VTP	pre-divergence	early	4/21.1%	15/88.9%	19	freshwater:eGFP;marine:mCherry
VTP	post-divergence	early	0/0%	23/100%	23	freshwater:eGFP;marine:mCherry
VTP	pre-divergence	mid	2/9.1%	20/90.9%	22	freshwater:eGFP;marine:mCherry
VTP	post-divergence	mid	0/0%	36/100%	36	freshwater:eGFP;marine:mCherry
VTP	pre-divergence	late	26/32.5%	54/67.5%	80	freshwater:eGFP;marine:mCherry
VTP	post-divergence	late	21/19.4%	87/80.6%	108	freshwater:eGFP;marine:mCherry
DTP	pre-divergence	early	0/0%	13/100%	13	freshwater:mCherry;marine:eGFP
DTP	post-divergence	early	0/0%	24/100%	24	freshwater:mCherry;marine:eGFP
DTP	pre-divergence	mid	1/6.3%	15/93.7%	16	freshwater:mCherry;marine:eGFP
DTP	post-divergence	mid	0/0%	24/100%	24	freshwater:mCherry;marine:eGFP
DTP	pre-divergence	late	51/49.5%	52/50.5%	103	freshwater:mCherry;marine:eGFP
DTP	post-divergence	late	27/26.7%	74/73.3%	101	freshwater:mCherry;marine:eGFP
VTP	pre-divergence	early	0/0%	16/100%	16	freshwater:mCherry;marine:eGFP
VTP	post-divergence	early	0/0%	23 (2 Freshwater [8.7%], 21 Marine [91.3%])	23	freshwater:mCherry;marine:eGFP
VTP	pre-divergence	mid	0/0%	13/100%	13	freshwater:mCherry;marine:eGFP
VTP	post-divergence	mid	0/0%	16/100%	16	freshwater:mCherry;marine:eGFP
VTP	pre-divergence	late	35/35.7%	63/64.3%	98	freshwater:mCherry;marine:eGFP
VTP	post-divergence	late	10/10.3%	87 (1 Freshwater [1%], 86 [88.7%] Marine)	97	freshwater:mCherry;marine:eGFP

For each tooth field (dorsal or ventral pharyngeal tooth plate, DTP or VTP), stage (pre-divergence = <20 mm fish length, post-divergence = >20 mm fish length, tooth stage [early or middle (mid), see Methods], the number (N), percentage (%) of detected mesenchymal bias in expression are listed, along with total number of teeth and genotype of transgene.

Supplemental methods

Multiple fluorescent reporter transgenes were assembled using the methods and primers as described below. Component abbreviations below are as follows: *Hsp70l* = stickleback *Hsp70l* promoter (O'Brown *et al.* 2015); GAB = mouse tyrosinase insulator (Bessa *et al.* 2009); *Col2a1a* = *Col2a1a R2* enhancer (Dale and Topczewski 2011).

***Col2a1a* containing insulator construct #1**

***Col2a1a* enhancer/*Hsp70l*→mCh+GAB+eGFP←*Hsp70l*/*Bmp6* enhancer**

The components of GAB, eGFP, and *Hsp70l*/*Bmp6* enhancer were amplified using primers MDS126/136, MDS137/89, and MDS90/131 respectively. The amplicons were combined with a modified plasmid (pT2He, modified to contain only polyclonal sites) linearized with *Nde*I and *BamH*I as well as Gibson Assembly master mix (NEB #E2611L) and incubated following the manufacturer's protocol. The resulting plasmid was digested with *Nde*I and *Bsu36*I and the fragments for the second half, *Col2a1a* enhancer/*Hsp70l* and mCherry, were amplified with MDS138/139 and MDS140/141 respectively. The plasmid and amplicons were combined with Gibson Assembly master mix and incubated following the manufacturer's protocol.

Primer name	Primer sequence	description
MDS126	cagataggcccctaaggactagtcatatgCTCACTATAGGGCGAATGGAGCTC	GAB forward
MDS136	atgtggtatggctgatGCCGCCAGTGTGATGGATATC	GAB reverse
MDS137	ccatcacactggcggcATCAGCCATACCACATTTGTAGAGG	eGFP forward
MDS89	tgcagtcgacggtGGTCGCCACCATGGTGAG	eGFP reverse
MDS90	catggtggcgaccACCGTCGACTGCAGGAAAAAAAAAC	*Bmp6*+*Hsp70l* forward
MDS131	taaataaagattcattcaagatctggatccGAGAGCATCCGTCTTGTGGG	*Bmp6*+Hsp70l reverse
MDS138	acacaggccagataggcccctaaggCGCTCCTTGAGGGTTTGAG	*Col2a1a* enhancer+*Hsp70l* forward
MDS139	ggtggcgaccGTCGACTGCAGGAAAAAAAAAC	*Col2a1a* enhancer+*Hsp70l* reverse
MDS140	tgcagtcgacGGTCGCCACCATGGTGAG	mCh forward
MDS141	cattcgccctatagtgagcatatgATCAGCCATACCACATTTGTAGAGG	mCh reverse

Primers used to amplify components of the *Col2a1a*:mCherry;*Bmp6* tooth enhancer:eGFP insulator containing bicistronic construct

***Col2a1a* containing insulator construct #2**

***Col2a1a* enhancer/*Hsp70l*→eGFP+GAB+mCh←*Hsp70l*/*Bmp6* enhancer**

The assembly of the second *Col2a1a* containing bicistronic construct is nearly identical to the first. All steps are the same except primers MDS137/89 were used to amplify mCherry in the first assembly step and primers MDS140/141 were used to amplify eGFP in the second assembly step. Due to identical sequence at the transition from *Hsp70l* to mCherry/eGFP and at the 3' end of the SV40 polyA sequence for each reporter, the same primers can be used to amplify both off of the original reporter plasmids.

Primer name	Primer sequence	description
MDS126	cagataggcccctaaggactagtcatatgCTCACTATAGGGCGAATGGAGCTC	GAB forward
MDS136	atgtggtatggctgatGCCGCCAGTGTGATGGATATC	GAB reverse
MDS137	ccatcacactggcggcATCAGCCATACCACATTTGTAGAGG	mCh forward
MDS89	tgcagtcgacggtGGTCGCCACCATGGTGAG	mCh reverse
MDS90	catggtggcgaccACCGTCGACTGCAGGAAAAAAAAAC	*Bmp6*+*Hsp70l* forward
MDS131	taaataaagattcattcaagatctggatccGAGAGCATCCGTCTTGTGGG	*Bmp6*+Hsp70l reverse
MDS138	acacaggccagataggcccctaaggCGCTCCTTGAGGGTTTGAG	*Col2a1a* enhancer+*Hsp70l* forward
MDS139	ggtggcgaccGTCGACTGCAGGAAAAAAAAAC	*Col2a1a* enhancer+*Hsp70l* reverse
MDS140	tgcagtcgacGGTCGCCACCATGGTGAG	eGFP forward
MDS141	cattcgccctatagtgagcatatgATCAGCCATACCACATTTGTAGAGG	eGFP reverse

Primers used to amplify components of the *Col2a1a*:eGFP;*Bmp6* tooth enhancer:mCherry insulator containing bicistronic construct

***Bmp6* intron 4 enhancer containing insulator construct**

***Marine Bmp6* enhancer/*Hsp70l*→eGFP+GAB+mCh←*Hsp70l*/Freshwater *Bmp6* enhancer**

The first assembly step was the same as the previous two constructs, except the primer pair MDS90/131 was used to specifically amplify the freshwater *Bmp6* enhancer. Linearization of the plasmid and Gibson Assembly was completed as before. The resulting plasmid was digested with *Nde*I and *Bsu36*I and the fragments for the second half, Marine *Bmp6* enhancer/*Hsp70l* and mCherry, were amplified with MDS164/139 and MDS140/141 respectively. The newly digested plasmid and amplicons were combined with Gibson Assembly master mix and incubated following the manufacturer's protocol.

Primer name	Primer sequence	description
MDS126	cagataggcccctaaggactagtcatatgCTCACTATAGGGCGAATGGAGCTC	GAB forward
MDS136	atgtggtatggctgatGCCGCCAGTGTGATGGATATC	GAB reverse
MDS137	ccatcacactggcggcATCAGCCATACCACATTTGTAGAGG	eGFP forward
MDS89	tgcagtcgacggtGGTCGCCACCATGGTGAG	eGFP reverse
MDS90	catggtggcgaccACCGTCGACTGCAGGAAAAAAAAAC	Freshwater *Bmp6*+*Hsp70l* forward
MDS131	taaataaagattcattcaagatctggatccGAGAGCATCCGTCTTGTGGG	Freshwater *Bmp6*+Hsp70l reverse
MDS164	ctgaaacacaggccagataggcccctaagGAGAGCATCCGTCTTGTG	Marine *Bmp6* enhancer+*Hsp70l* forward
MDS139	ggtggcgaccGTCGACTGCAGGAAAAAAAAAC	Marine *Bmp6* enhancer+*Hsp70l* reverse
MDS140	tgcagtcgacGGTCGCCACCATGGTGAG	mCh forward
MDS141	cattcgccctatagtgagcatatgATCAGCCATACCACATTTGTAGAGG	mCh reverse

Primers used to amplify components of the Freshwater *Bmp6* tooth enhancer:eGFP;marine *Bmp6* tooth enhancer:mCherry insulator containing bicistronic construct

Scoring effectiveness of insulators

To assess insulator effectiveness, all surviving injected fish were raised to 7 days post fertilization. At this time point the *Bmp6* intronic enhancer drives robust reporter expression in multiple domains including the distal edges of the median and pectoral fins, while the *Col2a1a* enhancer drives expression in the notochord (Cleves *et al.* 2018; Erickson *et al.* 2016). Four anatomical domains were scored for insulator effectiveness: the left and right pectoral fins, the median fin, and the notochord. Insulator efficiency was scored on a scale of 0 (apparent complete lack of insulation) to 2 (fully insulated enhancers) for each domain in which expression was observed. Insulation activity was only assessed for domains in which expression of at least a single fluorophore was present. Since effectiveness was scored in F_0 fish which are mosaic for the injected transgene, not all domains expressed a fluorophore.

Supplemental Results

Insulator effectiveness in bicistronic constructs

Insulator scores were not significantly different across injection clutches for the *Col2a1a R2*:mCherry; *Bmp6* tooth enhancer:eGFP construct (Kruskal-Wallis left pectoral fin P = 0.075,

right pectoral fin $P = 0.52$, median fin fold $P = 0.116$, Wilcoxon rank sum notochord $P = 0.25$), nor the *Col2a1a R2*:eGFP; *Bmp6* tooth enhancer:mCherry construct (Wilcoxon rank sum left pectoral fin $P = 0.144$, right pectoral fin $P = 0.134$, median fin fold $P = 0.211$), suggesting that the inter-clutch variation did not have a significant impact on insulation scores. The left pectoral fin ($P = 0.036$) and the median fin fold ($P = 0.016$) were found to be significantly different between the two constructs while the right pectoral fin ($P = 0.68$) and notochord ($P = 0.29$) were not significantly different.

Marine enhancer activity in the epithelium differs across tooth stage and fish size

In post-tooth number divergence fish activity of the freshwater enhancer was observed in the epithelium in both ventral and dorsal tooth plates in all pre-eruption teeth (marine:mCherry;freshwater:eGFP ventral: 59/59, dorsal: 44/44, and marine:eGFP;freshwater:mCherry ventral: 39/39, dorsal: 48/48), while the marine allele was observed in a subset of pre-eruption teeth (marine:mCherry;freshwater:eGFP ventral: 50/59 [84.7%], dorsal: 33/44 [75.0%], and marine:eGFP;freshwater:mCherry ventral: 35/39 [89.7%], dorsal: 34/48 [70.8%]). A higher percentage of early stage pre-eruption germs had marine activity in the epithelium compared to middle stage pre-eruption germs (marine:mCherry;freshwater:eGFP ventral: 20/23 [87.0%], dorsal: 24/29 [82.8%], and marine:eGFP;freshwater:mCherry ventral: 21/23 [91.3%], dorsal: 18/24 [75%]) than middle stage germs (marine:mCherry;freshwater:eGFP ventral: 30/36 [83.3%], dorsal: 9/15 [60.0%], and marine:eGFP;freshwater:mCherry ventral: 14/16 [87.5%], dorsal: 16/24 [66.7%]). In contrast to post-divergence, or > 20 mm total length, the marine enhancer in pre-divergence fish

was active in every pre-eruption tooth germ (marine:mCherry;freshwater:eGFP ventral: 31/31, dorsal: 36/36, and marine:eGFP;freshwater:mCherry ventral: 29/29, dorsal: 29/29).

Ventral bias of evolved enhancer shifts

Quantification of epithelial and mesenchymal expression, and bias towards enhancer activity was scored for three tooth plates of each type (ventral and dorsal) at pre and post tooth number divergence. In post divergence fish, activity of the freshwater enhancer was observed in the epithelium in both ventral and dorsal tooth plates in nearly all pre-eruption teeth (Figure S6A & Table S3). The marine allele was detected in the epithelium of only a subset of pre-eruption teeth, from approximately 70-90% of pre-eruption teeth in pooled tooth plate data (Figure S6A). When combining tooth plate data for each genotype the marine enhancer was active in the epithelium in a higher percentage of early stage germs compared to middle stage (marine:mCherry;freshwater:eGFP early: 44/52 [84.6%], middle 39/51 [76.5%] and marine:eGFP;freshwater:mCherry early: 39/47 [83.0%], middle 30/40 [75%]). The pattern is still present when data is sorted by tooth plate and genotype (Supplemental Material). Therefore, while there does appear to be a stage effect, variation also exists within stages. Overall, the freshwater enhancer drove expression more frequently and more robustly in the epithelium of early and middle stage teeth compared to the marine allele in post divergence fish. However, in pre-divergence fish, the epithelium of all pre-eruption teeth exhibited robust expression of both enhancers, across both genotypes and tooth plates (Figure S6A).

A bias towards the marine allele in the mesenchyme was observed in nearly every early or middle stage tooth germ, while the lack of bias, or entirely overlapping mesenchymal expression, was almost exclusively observed in late stage (erupted) tooth germs (Table S4). The

ventral tooth plates had an increased prevalence of marine enhancer bias in the mesenchyme of individual teeth compared to the dorsal tooth plates (marine:mCherry;freshwater:eGFP ventral: 146/167 [87.4%], dorsal: 102/149, [68.5%] and marine:eGFP;freshwater:mCherry ventral: 123/136 [90.4%], dorsal: 122/149 [81.9%]). In early and middle stage teeth, we observed a consistent marine bias in the mesenchyme of both the ventral and dorsal tooth plates. In fully formed erupted teeth, a difference between the tooth plates became apparent. A larger proportion of erupted teeth were observed to have a marine bias in the mesenchyme in the ventral tooth plate compared to the dorsal tooth plate (Figure S6B-C).

There was a reduction in the proportion of erupted teeth with a marine bias when comparing post to pre divergence fish for all integrations and tooth plates (pre-divergence marine:mCherry;freshwater:eGFP ventral 54/80 [67.5%], dorsal 55/91 [60.4%] and marine:eGFP;freshwater:mCherry ventral 63/98 [64.3%], dorsal 51/103 [49.5%]) (Figure S6B) except for the dorsal tooth plates in the freshwater:eGFP;marine:mCherry genotype. Overall a bias towards marine expression in the mesenchyme was observed, with a consistently larger proportion of late stage teeth demonstrating a bias in the ventral teeth compared to the dorsal teeth, with the difference between tooth plates becoming more drastic in larger fish. Thus, the trend in marine mesenchymal bias across dorsal versus ventral tooth plates mirrors the chromosome 21 tooth number QTL, which had a 28 LOD greater effect on ventral pharyngeal tooth number than dorsal pharyngeal tooth number (Miller *et al.* 2014). In addition, the difference in bias between pre-divergence and post-divergence fish is consistent with allele specific expression data in which early in development the marine and freshwater alleles of *Bmp6* are expressed at more similar levels, while in older fish there is a *cis*-regulatory reduction in expression of the freshwater allele (Cleves *et al.* 2014).

Mesenchymal bias differs across tooth stage, plate, and fish size

Mesenchymal bias, in which one enhancer was observed to drive a broader domain within the mesenchyme, was scored for post divergence fish. In early and middle stage teeth, we observed a consistent marine enhancer bias in the ventral (marine:mCherry;freshwater:eGFP early: 23/23, middle: 36/36, marine:eGFP;freshwater:mCherry early: 21/23 [91.3%], middle: 16/16) and dorsal tooth plates (early: 28/29, 96.6%, middle:15/15, marine:eGFP;freshwater:mCherry early: 24/24, middle: 24/24)). A larger proportion of functional, erupted teeth were observed to have a marine bias in the mesenchyme in the ventral tooth plate (marine:mCherry;freshwater:eGFP 87/108 [80.6%], marine:eGFP;freshwater:mCherry 86/97 [88.7%]) compared to the dorsal tooth plate (marine:mCherry;freshwater:eGFP 59/105 [56.2%], marine:eGFP;freshwater:mCherry 74/101 [73.3%] (Figure S6B-C). There was a reduction in the proportion of erupted teeth with a marine bias when comparing post to pre divergence fish for all integrations and tooth plates (pre-divergence marine:mCherry;freshwater:eGFP ventral: 54/80 [67.5%] and marine:eGFP;freshwater:mCherry ventral: 63/98 [64.3%], dorsal pre: 51/103 [49.5%]) (Figure S6B) except for the dorsal tooth plates in the freshwater:eGFP;marine:mCherry genotype (pre: 55/91 [60.4%], post: 59/105[56.2%]).
